# Light‐Driven Reconfigurable Logic in a Monolithic Perovskite Device via Nonlinear Photoresponse Switching

**DOI:** 10.1002/adma.202509566

**Published:** 2025-11-05

**Authors:** Dante Ahn, Youngsoo Jang, Minz Lee, WooKyung Jeon, Yohan Yoon, Heon Lee, Assa Aravindh Sasikala, Namsoo Lim, Chandran Balamurugan, Hyeonghun Kim, Gun‐Young Jung, Sooncheol Kwon, Minah Seo, Yusin Pak

**Affiliations:** ^1^ Sensor System Research Center Korea Institute of Science and Technology (KIST) Seoul 02792 Republic of Korea; ^2^ KU‐KIST Graduate School of Converging Science and Technology Korea University Seoul 02841 Republic of Korea; ^3^ Department of Materials Science and Engineering Korea University Seoul 02841 Republic of Korea; ^4^ Department of Materials Engineering Korea Aerospace University Goyang 10540 Republic of Korea; ^5^ Research Unit of Sustainable Chemistry University of Oulu Oulu 90570 Finland; ^6^ Department of Physics Durham University Durham DH1 3LE UK; ^7^ Department of Energy and Materials Engineering Dongguk University‐Seoul Seoul 04620 Republic of Korea; ^8^ School of Chemical Engineering Chonnam National University Gwangju 61186 Republic of Korea; ^9^ Gwangju Institute of Science and Technology (GIST) School of Materials Science and Engineering Gwangju 61005 Republic of Korea; ^10^ Department of Physics Sogang University Seoul 04017 Republic of Korea; ^11^ Division of Advanced Materials Engineering Jeonbuk National University (JBNU) Jeonju 54896 Republic of Korea

**Keywords:** dual photogate, logic gate, non‐linear photoresponse, perovskite photodetector, photocurrent switching

## Abstract

Modulating nonlinear carrier dynamics in a single‐layer device is essential for achieving complex logic operations with minimal power consumption; however, it remains challenging due to inherently linear charge transport and unipolar photoresponses. Here, a multifunctional optoelectronic logic gate (OELG) based on a bias‐free, single‐layer perovskite device is reported that exhibits light intensity‐dependent polarity switching. Incorporation of poly‐L‐lysine into MAPbI_3_ enables trap‐state engineering for nonlinear response modulation. An asymmetric dual‐photogate architecture allows spatially controlled charge transport by tuning the position of incident light. This configuration enables the realization of all eight fundamental logic gate functions, including XOR and XNOR, in a single material and device. Additionally, the device independently handles two channels, amplitude inputs, and temporal modulation inputs. It performs logic operations not by pixel‐level imaging, but by applying a scenario‐based conceptual modulation map to the device, with the outputs derived from experimentally recorded photovoltage responses. These findings establish a promising platform for compact, energy‐efficient, light‐driven logic systems with potential applications in light fidelity (Li‐Fi) communication and on‐device artificial intelligence.

## Graphical abstract

This study demonstrates a monolithic perovskite OELG device that performs all eight logic operations, including XOR and XNOR, without external bias. Enabled by trap‐engineered MAPbI_3_:PLL and dual photogates, it achieves reconfigurable logic and parallel decoding of amplitude–frequency signals, supporting scenario‐configured logic‐level separation for sensor‐front‐end preprocessing.

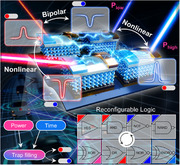

## 1 Introduction

Nonlinear photoresponses are key to advancing optoelectronic systems capable of performing complex tasks such as logic processing, data encryption, and real‐time visual object recognition under light stimuli.^[^ ^1^, ^2^, ^3^ ^]^ Recently, optoelectronic materials with high detectivity and in‐sensor processing have attracted increasing attention in advanced photodetector and neuromorphic applications.^[^ ^4^, ^5^ ^]^ However, in conventional semiconductors, photogenerated charge carriers typically follow fixed and symmetric transport paths, leading to predominantly linear photocurrent responses.^[^ ^6^, ^7^ ^]^ This intrinsic behavior fundamentally limits the realization of nonlinear functionalities, thus confining the development of higher‐order logic operations and dynamic signal processing at the device level.^[^ ^8^, ^9^, ^10^ ^]^

Achieving nonlinear photoresponses requires more than efficient light absorption; it demands careful engineering of both material properties and device architectures. Specifically, mechanisms such as charge transport asymmetry,^[^ ^11^ ^]^ trap‐assisted recombination,^[^ ^12^ ^]^ and field‐dependent carrier mobility must be modulated in tandem to enable light‐intensity‐dependent switching and nonlinear behavior.^[^ ^13^ ^]^ A number of promising approaches have been proposed to address this challenge. For example, light‐intensity‐induced photocurrent switching (LIIPS) in photoelectrochemical systems has been shown to reverse photocurrent polarity by controlling trap filling and carrier recombination dynamics.^[^ ^14^ ^]^ Similarly, core‐shell nanowires composed of nitrides and amorphous metal oxides have demonstrated wavelength‐dependent nonlinearities through photo‐induced redox reactions.^[^ ^11^ ^]^ Thermo‐optic modulation using plasmonic heating in Galinstan nanodroplets has enabled broadband spatial logic operations,^[^ ^15^ ^]^ while ferroelectric control in P(VDF‐TrFE)/MoS_2_ heterostructures has facilitated reconfigurable doping and photocurrent.^[^ ^16^ ^]^

Despite their functional merits, many of these strategies rely on extrinsic effects, such as electrochemical reactions, stacked layers of distinct light‐absorbing materials, or external circuit components, that increase fabrication complexity and reduce integration potential for scalable, energy‐efficient systems.^[^ ^17^ ^]^ As a result, the realization of compact, monolithic optoelectronic logic gate (OELG) devices with intrinsic nonlinear responses remains limited. Most reported OELG demonstrations are based on stacked layers of distinct light‐absorbing materials or modular device arrays, with single‐device implementations typically restricted to fewer than six logic functions.^[^ ^18^, ^19^, ^20^, ^21^, ^22^ ^]^ Notably, advanced logic operations such as XOR and XNOR, which require bipolar or polarity‐switchable photoresponses, have rarely been realized within a single‐layer optoelectronic platform.

In this study, we report a monolithic, bias‐free OELG based on a single‐layer perovskite photodetector composed of methylammonium lead iodide (MAPbI_3_) blended with the cationic polymer poly‐L‐lysine (PLL). The PLL interacts electrostatically with mobile ionic species (e.g., MA⁺, I^−^) within the perovskite lattice to form deep trap states, which enable intensity‐dependent modulation of photocurrent polarity.^[^ ^23^, ^24^, ^25^, ^26^ ^]^ Combined with an asymmetric dual‐photogate design, our device allows spatially tunable charge transport and nonlinear photoresponse within a single material system without the need for external bias or additional circuitry. As a result, all eight fundamental logic gate operations, including XOR and XNOR, are successfully demonstrated in a single device. Moreover, the device independently processes amplitude inputs and temporal modulation inputs through dual photogate channels to support scenario‐based logic gate operation at the device level. These results establish a compact and energy‐efficient platform for reconfigurable optoelectronic logic and parallel photonic signal processing.

## 2 Results and Discussion

**Figure 1:**
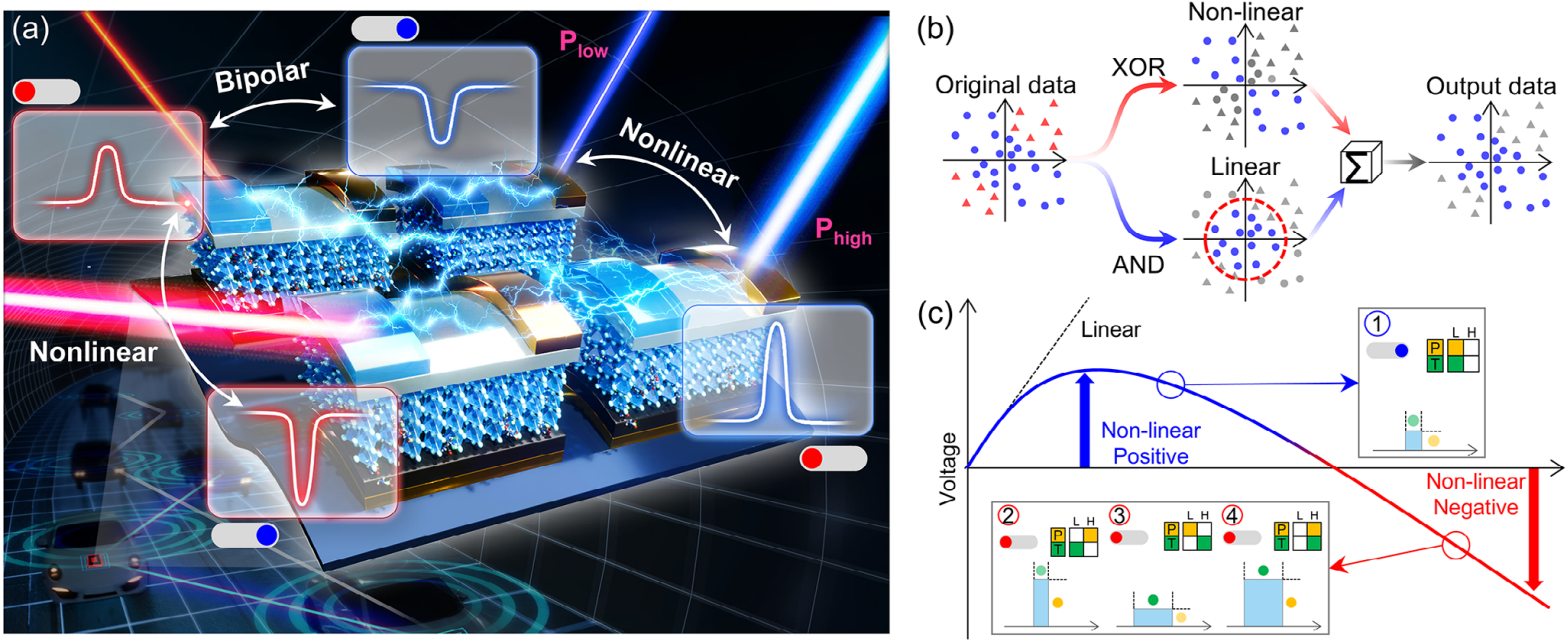
a) Conceptual illustration of a dual‐photogate perovskite OELG platform integrating nonlinear and bipolar photoresponse mechanisms within a monolithic architecture. Light‐intensity‐dependent trap filling enables dynamic control of photocurrent polarity, supporting reconfigurable logic operations. b) Schematic representation of nonlinear data separation using XOR and AND logic operations. c) Light‐programmable trap‐filling regimes governed by both intensity and spatial position, enabling the realization of all eight logic operations, including XOR and XNOR.

**Figure** **1** provides a conceptual overview of the device architecture and operating principle that enable fully reconfigurable and reprogrammable OELG operations, including nonlinear logic, within a single perovskite device. As shown in Figure 1a, the device integrates the light‐driven behavior of trap states into the perovskite active layer. By modulating trap filling and release through variations in light power, exposure time, and illumination location, the polarity of the photoresponse can be dynamically reversed. Simultaneous control of both bipolar and nonlinear photoresponses within a single semiconductor‐based photodetector has not been demonstrated to date. Figure 1b illustrates why nonlinear logic is essential for classifying complex datasets. While linear operations like AND are limited in distinguishing overlapping inputs, nonlinear functions such as XOR enable clear separation and advanced decision‐making capabilities within a single device. This functionality makes the system highly promising for emerging applications such as Li‐Fi communication, in‐device visual signal processing, and secure optical computing, where adaptive, light‐driven logic is key. To enable such nonlinear separation, simultaneous control of both bipolar and nonlinear photoresponses is essential. As shown in Figure 1c, the transition from linear to nonlinear behavior and the corresponding polarity switching can be precisely tuned by adjusting the aforementioned illumination conditions. This allows all eight fundamental OELGs, including XOR and XNOR, to be implemented within a monolithic device, with no need for structural reconfiguration or external electrical bias.

**Figure 2:**
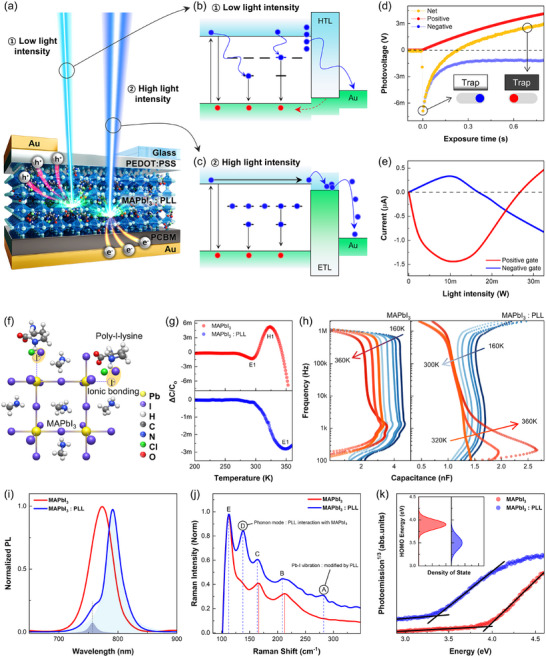
a) Layered device structure: Au/PEDOT:PSS/MAPbI_3_:PLL/PCBM/Au. Schematic diagrams of charge extraction pathways under (b) low and (c) high light intensities. d) Photovoltage reversal behavior attributed to trap filling under prolonged low‐intensity illumination. e) Polarity switching is controlled by light intensity and photogate position. f) Schematic of iodine vacancy formation and ionic interaction via PLL blending. g) DLTS spectra showing only shallow traps (E_1_ and H_1_) in pristine MAPbI_3_ and a deep trap (E_2_) in MAPbI_3_:PLL. h) Temperature‐dependent capacitance profiles highlighting nonlinear carrier dynamics associated with deep traps. i–k) Spectroscopic validation of trap states using (i) PL red‐shift and broadening, (j) Raman spectral shifts with PLL‐induced vibrational modes, and (k) UPS edge broadening, indicating increased energetic disorder due to PLL doping.

To investigate the mechanism behind LIIPS, we designed a device with a layered structure of Au/poly(3, 4‐ethylenedioxythiophene):poly(styrenesulfonate(PEDOT:PSS, hole transport layer)/MAPbI_3_:PLL/[6, 6]‐phenyl‐C₆₁‐butyric acid methyl ester (PCBM, electron transport layer)/Au (**Figure** **2**a), using a 105 µm‐diameter optical fiber mounted on a customized probe for precise control of light intensity and position (Figure S1, Supporting Information). The LIIPS mechanism operates by dynamically switching the direction of photocurrent (polarity) through the filling and emptying of trap states within the perovskite in response to varying light intensity and exposure time. Under low light intensity (Figure 2b), photogenerated electrons are captured by the deep traps, suppressing the forward current. As the light intensity increases (Figure 2c), these traps gradually saturate, releasing carriers that restore forward charge transport. Time‐resolved photovoltage measurements under low‐intensity illumination (Figure 2d; Figure S2, Supporting Information) confirmed that progressive trap filling reverses the net photovoltage from negative to positive polarity, with the transition occurring at 0.217 s. Once the traps are saturated, the net photovoltage recovers to its original positive polarity, and this switching behavior is further supported by current measurements as a function of light intensity (Figure 2e). Compared to the dark condition, when light is applied to the positive gate, the current decreases to −1.47 µA under illumination intensities below 26 mW and increases to +0.47 µA above that threshold (Figure S3, Supporting Information). These results demonstrate that, by modulating both light intensity and illumination location, four distinct current states characterized by polarity (positive or negative) and temporal slope (increasing or decreasing) can be selectively accessed, thereby enabling reconfigurable OELG operations.

The origin of the trap states can be attributed to electronic‐level modifications introduced by PLL incorporation into MAPbI_3_ (Figure 2f). Deep‐level transient spectroscopy (DLTS) confirmed that PLL doping leads to the emergence of trap states, possibly associated with modified iodine vacancies or polymer‐induced defects. These trap states significantly alter the charge extraction pathway. As shown in Figure 2g, only shallow traps were identified in pristine MAPbI_3_ (E_1_ at E_c_−0.33 eV, likely due to iodine vacancies, and H_1_ at E_v_+0.41 eV, attributed to MA^+^‐related defects), whereas MAPbI_3_:PLL exhibited a deep trap state (E_2_) at E_c_−0.88 eV (Figure S4, Table S1, Supporting Information). Here, E_c_ and E_v_ denote the conduction band minimum and valence band maximum, respectively, while E, H correspond to negatively charged and positively charged defects. Temperature‐dependent capacitance measurements^[^ ^27^ ^]^ further revealed nonlinear charge dynamics associated with the deep traps (Figure 2h). In pristine MAPbI_3_, capacitance continuously decreased with increasing temperature across all frequencies, except for a slight increase near 1 kHz. In contrast, MAPbI_3_:PLL showed a non‐monotonic response, with capacitance decreasing at temperatures below 300 K and starting to increase above 320 K at low frequencies. This reversal is attributed to thermally excited carriers escaping from deep traps and accumulating at the interfaces, which narrows the band bending and enhances the interfacial capacitance.

To further validate the presence of trap states, we conducted spectroscopic analyses. Photoluminescence (PL) spectra (Figure 2i) exhibited a red‐shifted emission from 770 nm in pristine MAPbI_3_ to 790 nm in MAPbI_3_:PLL, accompanied by spectral broadening and the emergence of a new peak at 760 nm.^[^ ^28^ ^]^ These features indicate enhanced nonradiative recombination via trap states, which is also supported by the reduced light absorption after the PLL doping (Figure S5, Supporting Information). Raman spectroscopy (Figure 2j) revealed distinct changes in phonon modes, including the appearance of additional vibrational peaks and shifts in the Pb–I lattice modes after the PLL doping.^[^ ^29^ ^]^ These results suggest that PLL incorporation induces localized lattice distortions around halide sites, as further supported by GIWAXS data showing decreased diffraction intensity at q ≈ 1.4 Å^−1^ (Figure S6, Supporting Information). Ultraviolet photoelectron spectroscopy (UPS, Figure 2k) of the PLL‐doped sample showed a broadened and downshifted highly occupied molecular orbital (HOMO) edge, shifting from 3.9 to 3.4 eV based on the extrapolation of the linear region. This shift, along with the spectral broadening, reflects an increased density of localized states near the valence band edge, indicating polymer‐induced energetic disorder.^[^ ^23^ ^]^ Surface morphology comparison is provided in Figure S7 (Supporting Information). These findings are consistent with DFT simulations (Figure S8, Supporting Information), which reveal that PLL doping introduces localized defect states near the band edges, primarily associated with iodine‐related lattice distortions.

**Figure 3:**
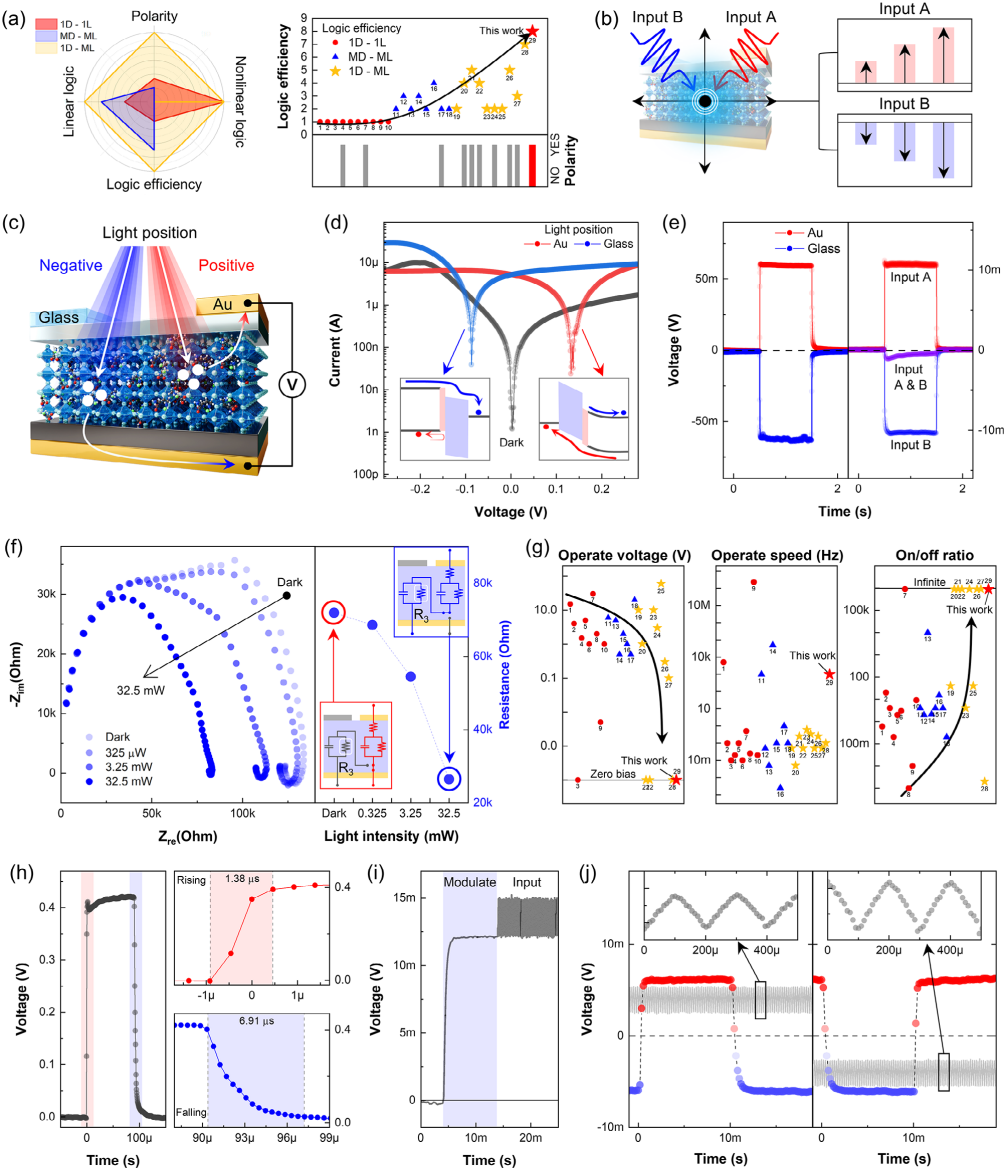
a) Classification of previously reported OELG devices into 1D‐1L, MD‐ML, and 1D‐ML categories. b) Schematic of position‐dependent bipolar photoresponse via dual photogate illumination. c) Asymmetric device structure showing short and long charge extraction paths at the Au and glass sides, respectively. d) Position‐dependent photocurrent response under selective photogate illumination. e) Photovoltage responses under individual (left) photogates and simultaneous (right) photogate illumination. f) Impedance spectroscopy showing semicircles (arcs) under negative photogate illumination and corresponding resistance changes. g) Comparative summary of operating voltage, speed, and on/off ratio for previously reported OELG devices. h) Rise and fall time measurements under 1 kHz switching. i) Independent output responses to simultaneous continuous and pulsed illumination. j) Output voltage behavior under mixed illumination: (left) positive response under negative standby, (right) negative response under positive standby, both illustrating frequency‐dependent polarity switching.

**Figure** **3**a classifies previously reported OELG devices into three categories: one‐device‐one‐logic (1D‐1L), multi‐device‐multi‐logic (MD‐ML), and single device multi logic (1D‐ML), summarizing the number of logic functions implemented in each.^[^ ^30^ ^]^ In recent studies, the 1D‐ML configuration has been the most frequently explored, where up to six logic gates have been demonstrated based on bipolar photoresponse enabled by additional photogating.^[^ ^31^, ^32^ ^]^ In this study, we realized eight distinct logic operations within a single perovskite device, with efficiency defined as the number of logic operations achieved per device. To achieve the full logic functionality, both mechanisms, position‐dependent bipolar photoresponse and intensity‐dependent nonlinear switching, were simultaneously utilized, as conceptually illustrated in Figure 3b. Here, the output polarity is determined by the combined effects of light power and exposure time: low light intensity and short duration, insufficient trap filling maintains the negative polarity (blue), whereas increasing either parameter leads to polarity switching toward the positive direction (red).

Light illumination near the Au electrode (positive photogate) induces a short vertical extraction path, while illumination near the glass side (negative photogate) results in a longer path, as described with arrows (Figure 3c). This asymmetric structure allows position‐dependent dual photogating in extraction length, leading to photocurrent responses with opposite polarities. As shown in Figure 3d, the photocurrent strongly depends on the illumination position. Light applied to the positive photogate causes hole extraction through PEDOT:PSS and MAPbI_3_ to the top electrode. In contrast, illumination at the negative photogate induces electron extraction through MAPbI_3_ and PCBM toward the bottom electrode, while hole transport is suppressed. This bipolar photoresponse arises from the material asymmetry between the Au and ITO/glass photogates, despite sharing the same bottom Au electrode. Figure 3e shows photovoltage responses under separate illumination at each photogate (left) and simultaneous illumination at both photogates (right). In the latter case, the output signal is cancelled as a result of asymmetric charge extraction. To investigate spatial dependence, light position was varied using an optical fiber, as shown in Figure S9 (Supporting Information). This bipolar response was consistently observed across 10 distinct devices (Figure S10, Supporting Information), and maintained over 10000 cycles with 10% degradation (Figure S11, Supporting Information).

Selective modulation of the output by varying the relative intensity and position is clearly supported by the impedance spectroscopy results in Figure 3f. Under illumination at the negative photogate, two arcs appear in the Nyquist plot. As the light intensity increases, the high‐frequency arc decreases in size, indicating reduced impedance. In this condition, the equivalent series resistance (R3) drops from 72 to 26 kΩ (Figure S12, Supporting Information). In contrast, illumination at the positive photogate produces a single arc, which gradually decreases in size with increasing light intensity (Figure S13, Supporting Information). This contrast between the two photogates reflects the difference in charge extraction paths depending on the illumination location.

Figure 3g summarizes the performance metrics of previously reported OELG devices, corresponding references listed in **Table** **1**. Most of these devices require a large external bias exceeding 10 V to enable optoelectronic logic operations. In contrast, our PLL‐doped perovskite device operates solely under light without any applied bias (i.e., zero bias). Moreover, it exhibits a reliable bipolar response without relying on stacked layers of distinct light‐absorbing materials. This configuration enables high‐speed operation at a frequency of 1 kHz, with measured response times of 1.38 µs (rise) and 6.91 µs (fall), as shown in Figure 3h. The fast and stable rise and fall behavior allows pulsed light to be used as an additional input signal. As demonstrated in Figure 3i, the device generates independent outputs under simultaneous exposure to continuous (modulate) and pulsed (input) light, without signal interference or cancellation. Figure 3j shows the output response under continuous light combined with pulsed inputs at 50 and 5000 Hz. In the left graph of Figure 3j, a 50 Hz pulsed input with relatively long off intervals results in a transition from positive to negative voltage. In contrast, under 5000 Hz illumination, the short pulse intervals cause charge accumulation, maintaining a positive polarity. This frequency‐dependent polarity retention and switching behavior suggests that the dual‐gated perovskite device can preserve and process mixed input signals without loss, even in high‐frequency operation regimes.

**Table 1:** Summary of the performance metrics of recently reported OELG devices, including operating voltage, switching speed, on/off ratio, number of logic functions, and device configuration.

Num	Refs.	Bipolar	Logic efficiency	Linear logic operation	Nonlinear logic operation	On/off ratio	Operation speed [Hz]	Operating voltage [V]
1	[33]	0	1	3	0	0.6	5000	15
2	[34]	0	1	3	0	20	0.1	4
3	[35]	0	1	1	0	4	0.01	0
4	[36]	1	1	1	0	0.2	0.02	1.5
5	[37]	0	1	2	0	2	0.1	5
6	[38]	0	1	0	1	3	0.01	1
7	[39]	1	1	0	1	infinite	0.5	30
8	[40]	0	1	1	0	0.001	0.025	2
9	[19]	0	1	2	1	0.01	2.3 × 10^8^	0.005
10	[41]	0	1	1	0	9	0.02	1
11	[42]	0	2	2	0	4	1000	6
12	[43]	0	3	3	0	2	0.05	−5
13	[44]	0	2	2	0	10 000	0.005	5
14	[45]	0	3	3	0	2.142	50 000	0.5
15	[46]	0	2	2	0	4	0.1	2
16	[47]	0	4	4	0	15	0.00025	1
17	[48]	1	2	2	0	4	1	0.5
18	[49]	0	2	2	0	0.2	0.1	20
19	[50]	0	2	2	0	40	0.05	10
20	[51]	1	4	4	0	Infinite	0.005	1
21	[52]	1	5	5	0	Infinite	0.25	0
22	[53]	1	4	4	0	Infinite	0.05	40
23	[54]	0	2	2	0	4	0.6	10
24	[55]	1	2	2	0	Infinite	0.33	3
25	[56]	0	2	2	0	40	0.05	60
26	[57]	1	5	5	0	Infinite	0.25	0.3
27	[58]	1	3	2	1	Infinite	0.05	0.1
28	[15]	0	7	5	2	0.002	0.1	0
29	This work	1	8	6	2	Infinite	1000	0

**Figure 4:**
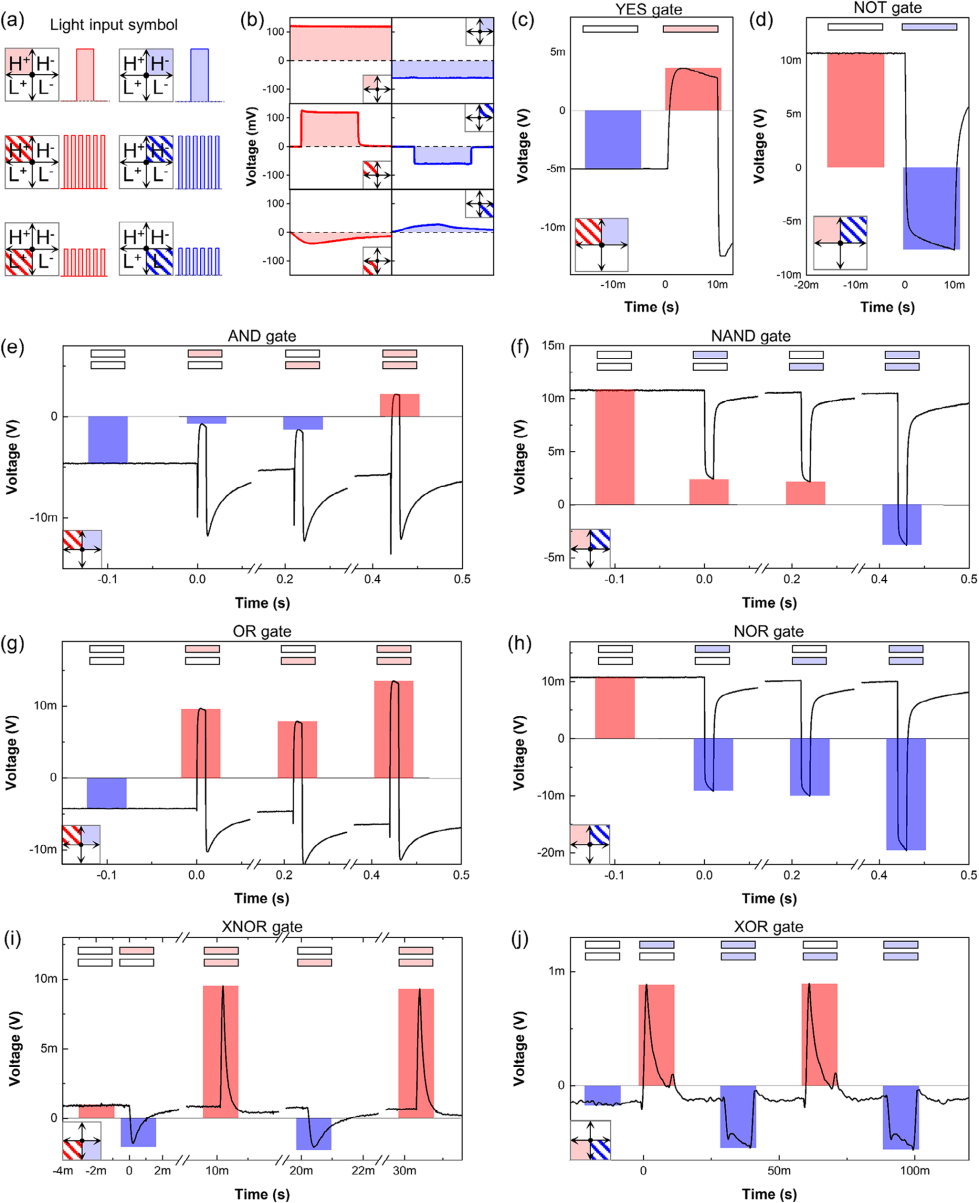
a) Definition of input conditions based on light type (continuous and pulsed), intensity (high or low), and photogate position (positive or negative). b) Output voltages corresponding to each illumination condition. c) YES (buffer) and d) NOT logic gate operations. e–h) Linear logic gates: AND, NAND, OR, and NOR. i–j) Nonlinear logic gates: XNOR and XOR.

**Figure** **4** presents all eight OELGs operated in the single perovskite device. As illustrated in Figure 4a, the input conditions are defined using four quadrants. Light is applied to the negative photogate in quadrants 1 and 4, and to the positive photogate in quadrants 2 and 3. Quadrants 1 and 2 represent high‐intensity illumination, while quadrants 3 and 4 represent low intensity. Striped regions indicate pulsed light inputs. The corresponding output voltages for each input configuration are shown in Figure 4b. A light input is defined as logic “1” and no light as logic “0”. A positive output voltage is interpreted as logic “1”, and a negative voltage as logic “0”. Figure 4c demonstrates a YES (buffer) gate, where simultaneous continuous and pulsed illumination on the respective photogates produces a positive output. Reversing the illumination positions inverts the polarity, implementing a NOT gate (Figure 4d).

By appropriately selecting the light intensity and spatial position of continuous and pulsed inputs, other logic gates such as AND, NAND, OR, NOR, XNOR, and XOR can be realized (Figure 4e–j). The illumination parameters for each logic gate, including wavelength, intensity, and pulse duration, are summarized in Table S2 (Supporting Information). When continuous light is applied to the negative photogate and pulsed light to the positive photogate, the device performs additive logic operations such as YES, AND, OR, and XNOR. Conversely, reversing the input configuration enables inversion or exclusive logic operations such as NOT, NAND, NOR, and XOR. Notably, the XNOR operation yields a positive output for inputs ‘0,’ and ‘1,1’, and a negative output for ‘0,1’, ‘1,0’, depending on whether the light intensity at each photogate is below or above the polarity switching threshold. XOR operates similarly but with the photogates spatially swapped. Repeated operation of the XNOR and XOR gates over 100 cycles is demonstrated in Figure S14 (Supporting Information). These results confirm that all eight fundamental logic operations can be dynamically reconfigured in a single device via optical modulation, without requiring complex circuit architecture or external biasing. Furthermore, sequential dynamic switching among linear and nonlinear logic gates—including AND, OR, NAND, NOR, XNOR, and XOR was demonstrated in Figure S15 (Supporting Information). Execution of all eight logic functions requires precise control of optical input position and intensity, which in practice may be achieved through automated, software‐controlled input systems.

**Figure 5:**
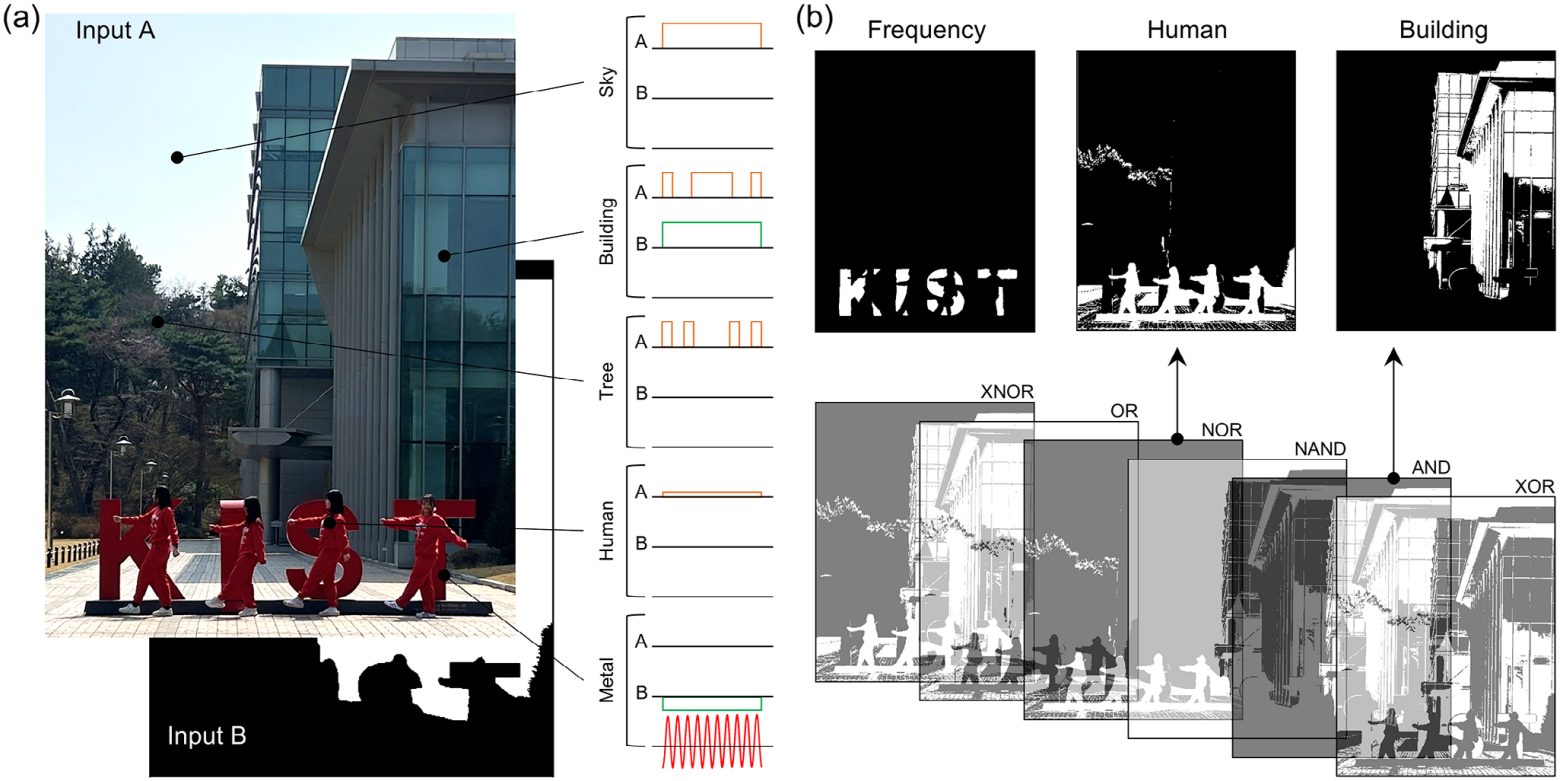
a) Input maps for a representative scene with temporally modulated illumination, pedestrian, tree, building, and sky. Input A is a binary intensity map derived from a grayscale camera image, while Input B is a scenario‐based conceptual binary map indicating whether temporal modulation is preserved or lost, and was not obtained at the pixel level. b) Device outputs obtained from experimentally measured photovoltage (Figure S17, Supporting Information), binarized with respect to 0 V, showing logic‐level operations that separate amplitude‐sensitive and frequency‐sensitive channels.

**Figure** **5** presents a scenario‐based proof‐of‐concept demonstration using our multifunctional OELG. Objects in the real scene of Figure 5a and their designated optical modulation characteristics were encoded into binary values (1 and 0), and the resulting bit information was optically fed into our perovskite OELG. The device executes eight Boolean logic operations, distinguishing between intensity‐sensitive and frequency‐sensitive domains when processing mixed inputs.

Input A is a binary intensity map obtained by thresholding a grayscale image of 1120 by 850 pixels. Input B is defined as a conceptual modulation map reflecting phase‐comparison principles, indicating whether temporal modulation of incident light is preserved or lost, and it was not obtained from real pixel‐level measurements. This map was encoded into binary values (‘1’ and ‘0’) and optically delivered to the device. The dual photogate design in Figure 3i,j processes the two domains independently, enabling interpretation of rapidly modulated light and slowly varying signals (Figure S16, Supporting Information). Input B was not measured at the pixel level in this work; however, under conditions including a stable modulated light source, a pixel‐level timing reference, and circuits for ambient‐light and noise suppression, Input B could be obtained under appropriate conditions.

As mentioned above, Inputs A and B, obtained respectively from a thresholded real image and a conceptual scenario map, were optically delivered to our OELG device through optical fibers, and the logic operations were performed based on the experimentally measured photovoltage (Figure S17, Supporting Information). Figure 5b shows the binarized outputs of these responses, where scene elements such as sculpture, pedestrian, tree, building, and sky are represented. For example, static objects can preserve the modulated signal so that it appears in the high‐frequency domain, whereas dynamic objects can induce phase variations that weaken the modulation so that it may no longer remain as a valid component. Inputs A and B were optically encoded and delivered to the device, which then performed Boolean logic operations, including NOR, AND, and XOR, to illustrate that distinct features can be differentiated at the device level. These results do not imply array‐level perception or selective isolation of specific objects, but rather demonstrate, under temporally modulated illumination, the proof‐of‐concept feasibility of logic‐level operation in a perovskite optoelectronic logic gate.

Practical use requires a stabilized modulated light source and detection synchronized to a reference clock, which enables frequency separation and suppression of ambient light. Under these conditions, per‐pixel demodulated information or modulation flags could serve as Input B. The OELG performs device‐level logic by combining intensity and frequency channels through a nonlinear bipolar photoresponse at zero external bias. Next steps include array scaling, synchronization and timing distribution, co‐design of optics, circuits, and packaging, encapsulation and environmental stabilization, and standardized verification of speed, power, and robustness. Meeting these requirements would allow OELG‐based logic to operate as a low‐power, high‐speed front‐end selector that reduces downstream processing load, providing a potential path toward deployment (Tables S3–S5, Supporting Information).

## 3 Conclusion

In summary, we demonstrated a monolithic, bias‐free perovskite logic device capable of performing all eight OELG operations, including XOR and XNOR. This achievement was made possible through two key strategies: the use of MAPbI_3_ blended with poly‐L‐lysine (PLL) to induce trap‐mediated nonlinear responses, and an asymmetric dual photogate architecture that enables precise bipolar modulation of charge transport via position‐ and intensity‐controlled illumination, without relying on stacked layers of distinct light‐absorbing materials or external bias. As a result, seamless switching between linear (YES, AND, OR, NOR, NOT, and NAND) and nonlinear (XOR and XNOR) logic gates was successfully realized within a single‐layer perovskite device for the first time. Additionally, simultaneous amplitude‐ and frequency‐based signal decoding enables independent handling of mixed optical inputs. In a conceptual vision proof‐of‐concept demonstration, the OELG performed multiple logic operations using a conceptual and an intensity map, demonstrating the feasibility of distinguishing different features. These results position our device as a compact and energy‐efficient platform for reconfigurable optoelectronic logic and parallel optical signal processing. While classical photodetector metrics such as detectivity and signal‐to‐noise ratio are important for practical deployment, their optimization lies beyond the current scope and will be explored in future work.

## 4 Experimental Section

### Fabrication of the MAPbI_3_ Device

A dual‐gate photogate device was fabricated on a substrate featuring alternating regions of glass and indium tin oxide (ITO). The fabrication process began with the spin‐coating of PEDOT:PSS at 5000 rpm for 40 s. Subsequently, a PbI_2_:PLL precursor solution (1 m in a DMF:DMSO mixture with a volume ratio of 0.8:0.2, containing 0.3 mg of PLL) was deposited at 2000 rpm for 20 s. The film was then converted to MAPbI_3_:PLL by spin‐coating a 0.25 m MAI solution in isopropanol at 3000 rpm for 30 s. A PCBM layer (0.044 m in chlorobenzene) was applied by spin‐coating at 2000 rpm for 20 s. Finally, a gold electrode was deposited by thermal evaporation, with electrical contact established via electrical connection to the adjacent ITO region.

### Thin Film Characterization

Ultraviolet photoelectron spectroscopy (UPS) was conducted using a Nexsa system (ThermoFisher Scientific) equipped with a He‐I (21.22 eV) source. Measurements were performed under a base pressure of 2.0 × 10^−8^ mBar with a beam size of 1.0 mm. The work function was determined at a pass energy of 1.0 eV with an applied bias of −10 V. UV–vis absorbance spectra were obtained using a DH‐2000 spectrometer (Ocean Insight). Raman spectroscopy was performed using a 532 nm laser on a Renishaw system, with a 10‐s exposure time and 10% laser power. Grazing‐incidence wide‐angle X‐ray scattering (GIWAXS) measurements were carried out at the Pohang Accelerator Laboratory using synchrotron radiation at an incidence angle of 1.5°. Photoluminescence (PL) spectra were measured using a Fluorolog‐3 system (HORIBA Jobin Yvon) with an excitation wavelength of 325 nm and a detection range of 400–800 nm at room temperature.

### Electrical Characterization

Impedance spectra were obtained using a µAUTOLAB FRA2 TYPE III system from 0.1 Hz to 100 kHz with a 0.01 V AC signal in potentiostatic mode, and fitted using NOVA software. Light sources were controlled with a MIGHTEX Universal LED Controller connected to four optical fibers. Current–voltage characteristics under varying light intensities were measured with a Keithley 2636B SourceMeter, while open‐circuit voltages were recorded using an RTM3004 oscilloscope (ROHDE & SCHWARZ). Under zero‐bias conditions, the output was measured as open‐circuit voltage using an oscilloscope.

### DLTS Measurement

Deep‐level transient spectroscopy (DLTS) measurements were conducted using a cryogenic probe station (Lakeshore TTPX) integrated with an impedance analyzer (Zurich Instruments MFIA). Temperature‐dependent scans were performed from 100 to 395 K in 1 K increments, averaging ten measurements per step to enhance signal reliability. Capacitance–voltage (C–V) profiles were acquired at 510 kHz to evaluate the doping density and depletion width. For DLTS analysis, a voltage pulse from −0.5 to 0 V was applied with a pulse duration of 1 s and a period of 5 s. DLTS characterizes deep‐level defects by monitoring capacitance transients induced by voltage pulses perturbing the steady‐state depletion region. The subsequent thermal emission of trapped carriers enables the extraction of defect parameters, including energy levels, capture cross‐sections, and concentrations, thereby providing critical insight into carrier recombination mechanisms in semiconductors.

## Conflict of Interest

The authors declare no conflict of interest.

## Supporting information



Supporting Information

## Data Availability

The data that support the findings of this study are available in the supplementary material of this article.
