# Supplementary material for: Light‐Driven Reconfigurable Logic in a Monolithic Perovskite Device via Nonlinear Photoresponse Switching
Source: Adv Mater. 2025 Nov 5;38(14):e09566. doi: 10.1002/adma.202509566 (PMC12966971; doi:10.1002/adma.202509566)
Supplement: Supplementary file 1 — Supporting Information [file ADMA-38-e09566-s001.docx]

Supporting Information

Light-Driven Reconfigurable Logic in a Monolithic Perovskite Device via Nonlinear Photoresponse Switching

Dante Ahn^1,2^, Youngsoo Jang^1,2^, Minz Lee^1,3^, WooKyung Jeon^4^, Yohan Yoon^4^, Heon Lee^3^, Assa Aravindh S^5,6^, Namsoo Lim^7^, Hyeonghun Kim^8^, Gun-Young Jung^9^, Sooncheol Kwon^7,*^_,_ Minah Seo^1,2,10*^ and Yusin Pak^1,11*^

^1^Korea Institute of Science and Technology (KIST), Sensor System Research Center, Seoul 02792, Republic of Korea

^2^Korea University, KU-KIST Graduate School of Converging Science and Technology, Seoul 02841, Republic of Korea ^3^Department of Materials Science and Engineering

^4^Korea Aerospace University, Department of Materials Engineering, Goyang 10540, Republic of Korea

^5^University of Oulu, Research Unit of Sustainable Chemistry, Oulu 90570, Finland

^6^Durham University, Department of Physics, Durham DH1 3LE, United Kingdom

^7^Dongguk University-Seoul, Department of Energy and Materials Engineering, Seoul 04620, Republic of Korea

^8^Chonnam National University, School of Chemical Engineering, Gwangju 61186, Republic of Korea

^9^Gwangju Institute of Science and Technology (GIST), School of Materials Science and Engineering, Gwangju 61005, Republic of Korea

^10^Sogang University, Department of Physics, Seoul 04017, Republic of Korea

^11^Jeonbuk National University (JBNU), Division of Advanced Materials Engineering, Jeonju 54896, Republic of Korea

********E-mail: kwansc12@dongguk.edu; mseo@kist.re.kr; yusinpak@jbnu.ac.kr*

**Keywords:** Perovskite photodetector, Logic gate, Non-linear photoresponse, Dual photogate, Photocurrent switching

**Supporting Information Contents**

1. Optical Fiber–Based Illumination Setup

2. Light-Induced Polarity Switching in Photovoltage

3. Nonlinear Photocurrent Characteristics in Asymmetric Dual Photogate Device

4. Deep Level Transient Spectroscopy (DLTS) and Trap State Analysis

5. Optical Characterization: Absorbance Spectra

6. Grazing Incidence Wide Angle X-ray Scattering (GIWAXS) Analysis

7. Surface SEM Image

8. Density Functional Theory (DFT) Simulation of Trap-Induced States

9. Spatial Mapping of Photovoltage Response

10. Device-to-Device Reproducibility

11. Endurance Test Under Repeated Illumination

12. Impedance Spectroscopy at the Negative Photogate

13. Impedance Spectroscopy at the Positive Photogate

14. Illumination Conditions for Logic Operations

15. Endurance of Light-Driven Nonlinear Logic Operations

16. Sequential and Dynamic Switching of Logic Operations

17. Analog Signal Separation for Parallel Logic Decoding

18. Analog Voltage Mapping Results Prior to Binarization

19. Commercial vision sensors and preprocessing strategies: a reference summary

**Section 1.** Optical Fiber–Based Illumination Setup


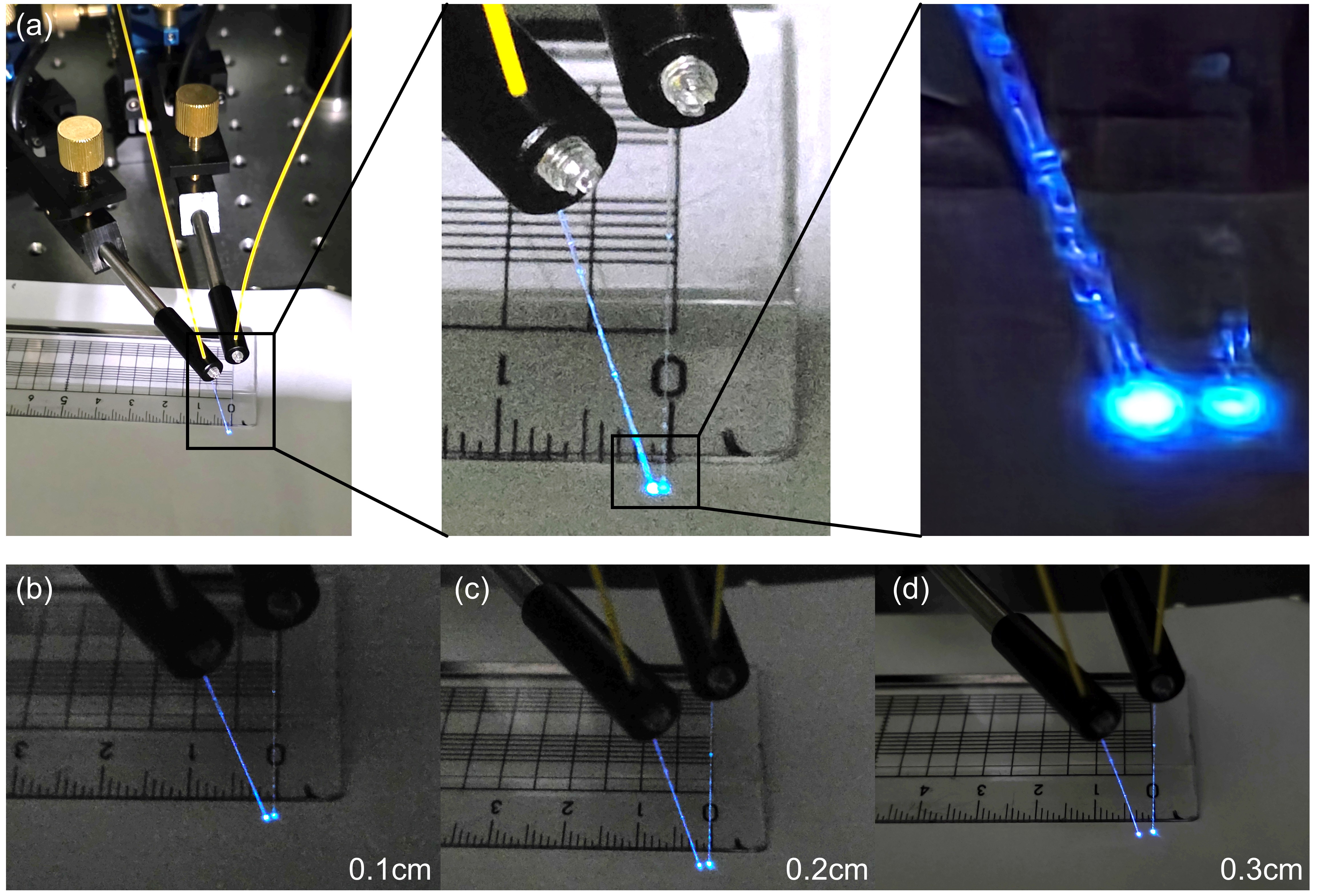


**Figure S1**. (a) Photographs of the customized electrical probe tip modified to hold an optical fiber. The same setup is shown at increasing magnifications, captured using a digital camera. (b–d) Camera images showing two optical fibers, with one fixed at position 0 cm and the other placed at (b) 0.1 cm, (c) 0.2 cm, and (d) 0.3 cm apart, respectively. The images demonstrate that light can be delivered to each photogate separately without mutual interference.

A 105 μm-diameter optical fiber was used to illuminate each photogate with minimal interference. The effective device area and the distance between photogates were both set to 0.2 cm to ensure spatial separation. A customized fiber-probe setup enabled precise manual alignment for reliable and reproducible illumination. To minimize optical interference, a fiber-coupled probe was positioned ~0.2 cm above the active area (diameter: 0.2 cm) to selectively illuminate each photogate.

**Section 2.** Light-Induced Polarity Switching in Photovoltage


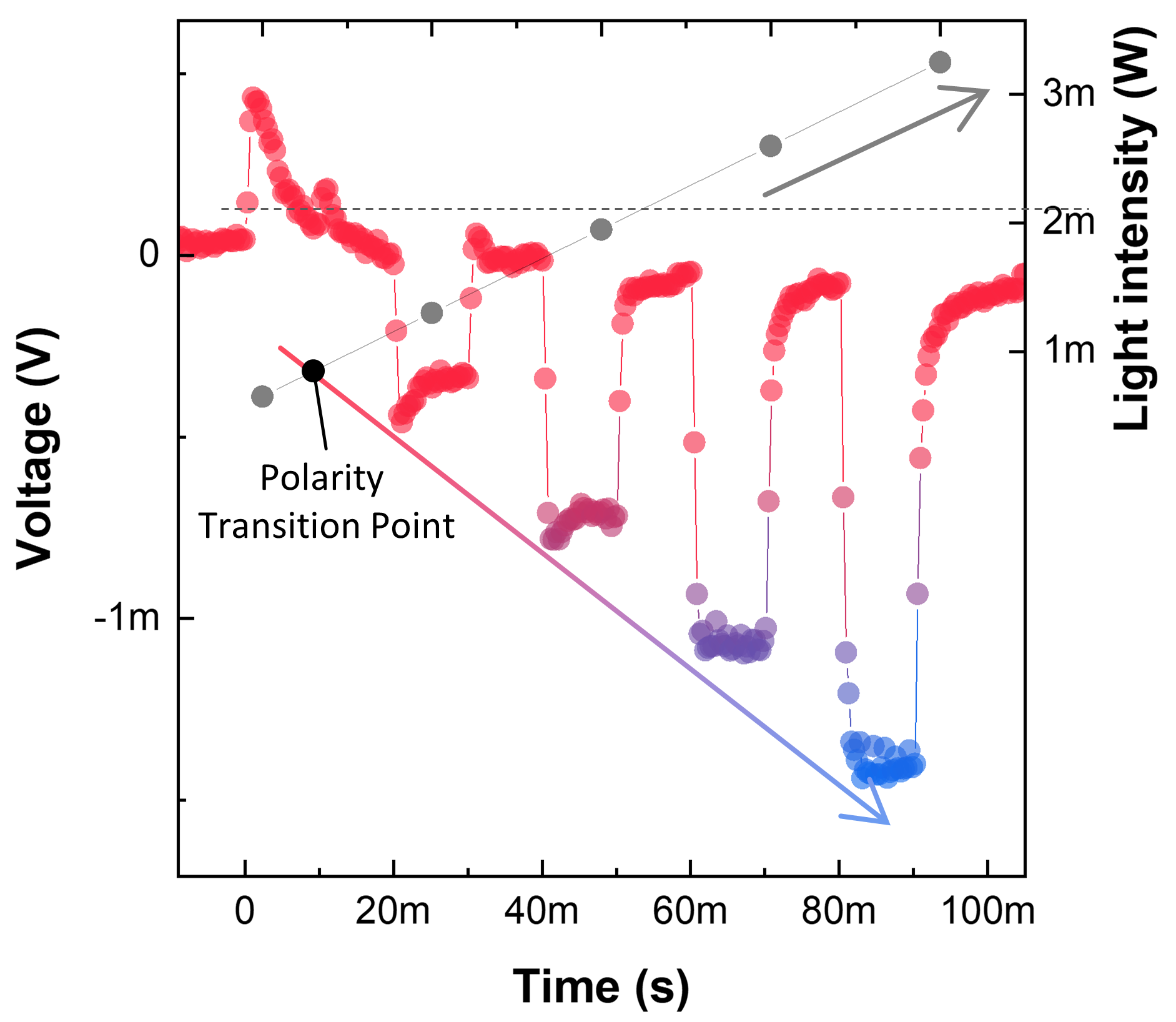


**Figure S2.** Polarity transition in photovoltage as a function of light intensity. The gray line-scatter plot indicates the applied light intensity, while the red and blue scatter points represent the output voltages, respectively, revealing a distinct transition in polarity with increasing light intensity.

**Section 3.** Nonlinear Photocurrent Characteristics in Asymmetric Dual Photogate Device


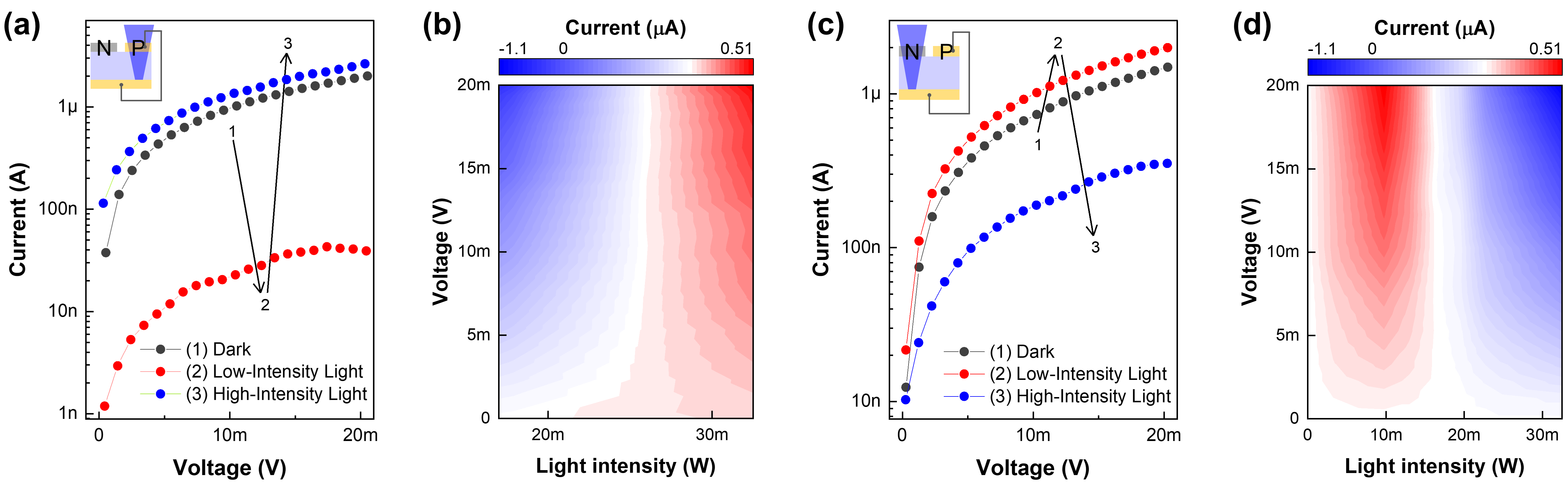


**Figure S3.** (a) Current–voltage characteristics of the asymmetric dual photo-gate device, measured with light applied to the positive photo-gate under dark (1), low-intensity (2), and high-intensity (3) conditions. (b) Current difference map of the positive gate, showing nonlinear increases in output current with increasing light intensity. (c) Current–voltage characteristics of the negative photo-gate under the same three conditions. (d) Current difference map of the negative gate, illustrating the opposite trend: current increases under low light but decreases beyond ~15 mW, indicating a polarity reversal in the current response.

Figure S3 presents the current–voltage characteristics of the dual photogate device under different light intensities, highlighting nonlinear and polarity reversing behaviors at each photogate. At the positive gate (Figure S3a and S3b), the photocurrent initially decreases and then increases nonlinearly beyond ~26 mW, indicating trap-mediated transport modulation. At the negative gate (Figure S3c and S3d) current increases at low light but decreases above ~15 mW, reflecting polarity reversal due to incomplete trap saturation. These complementary responses confirm spatially resolved nonlinear dynamics driven by local trap filling and carrier mobility modulation.

**Section 4.** Deep Level Transient Spectroscopy (DLTS) Measurement and Trap State Analysis


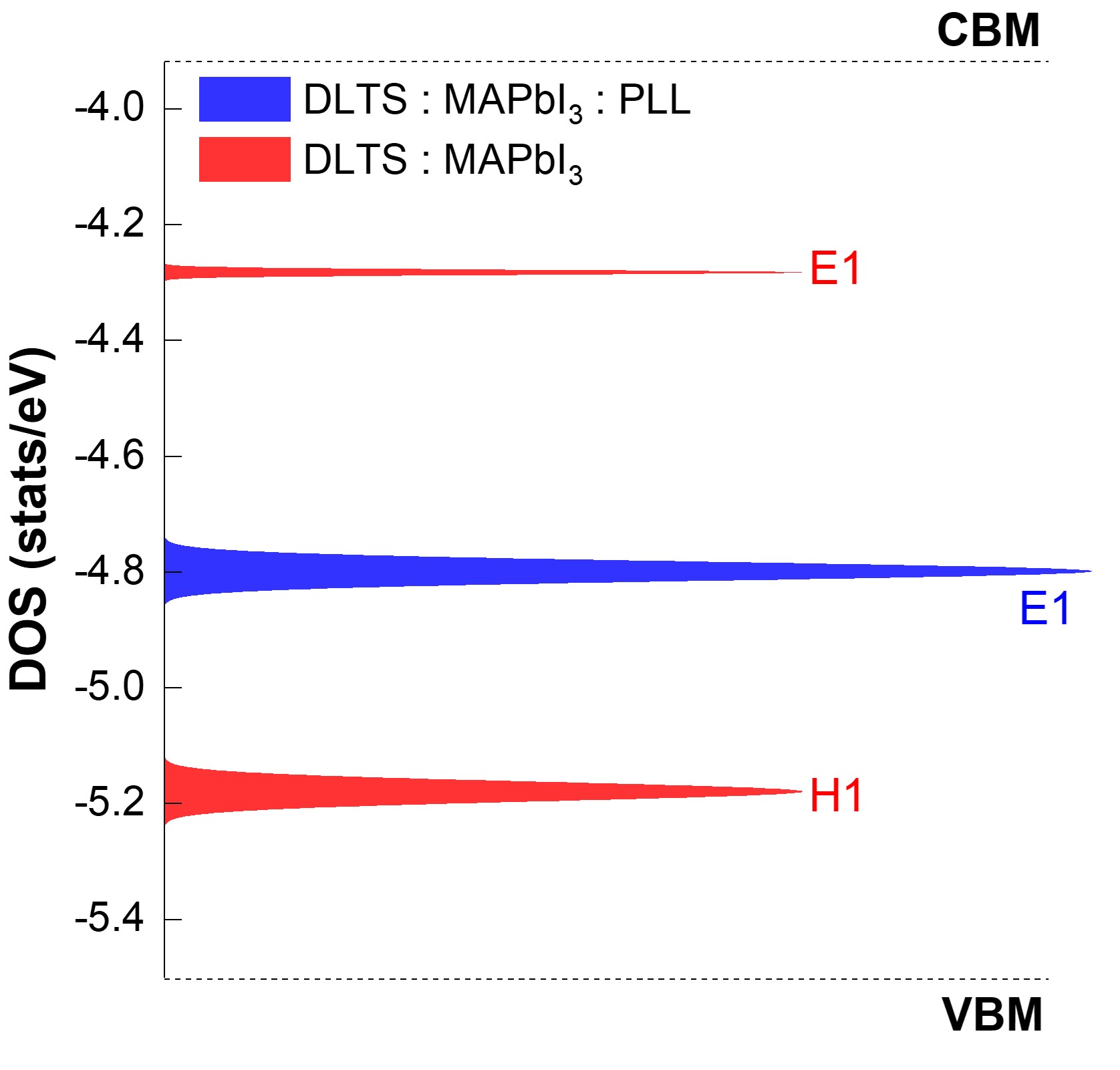


**Figure S4.** Trap state distributions within the band gap reconstructed from DLTS. Two traps were observed in pristine MAPbI_3_, whereas MAPbI_3_:PLL exhibited a single, deeper-level trap based on activation energies from DLTS peaks.

| Sample | Peak temperature (K) | Activation Energy E_a_ (eV) | Trap density N_T_ (cm^-3^) |
| --- | --- | --- | --- |
| MAPbI_3_ | E_1_ (293.81) | E_C_ - 0.33 | 3.35 x 10^14^ |
|  | H_1_ (324.23) | E_V_ + 0.41 | 1.32 x 10^15^ |
| MAPbI_3_:PLL | E_1_ (348.62) | E_C_ - 0.88 | 1.28 x 10^15^ |

**Table S1.** Trap activation energies and densities extracted from DLTS. Activation energies were obtained by Arrhenius fitting, and trap densities were calculated from capacitance transients and C–V data at 510 kHz.

Trap activation energies (E_a_) were extracted from the peak temperature (T_p_) of DLTS spectra using the Arrhenius relation, assuming single-carrier emission. Trap densities (N_T_) were calculated from the capacitance transients (∆C) relative to the steady-state capacitance (C_0_) using:

$$N_{t}=\frac{2\cdot\Delta C\cdot C_{0}}{q\cdot A\cdot W}$$

where q is the elementary charge, A is the device area, and W is the depletion width from C-V measurements at 510 kHz.

Figure S4 maps DLTS signal magnitude onto energy levels based on the extracted Ea, and detailed parameters are listed in Table S1. The pristine MAPbI_3_ sample revealed two traps:

- E_1_ at 293.8 K (E_a_ = E_c_ – 0.33 eV, N_t_ = 3.35×10¹⁴ cm⁻³)
- H_1_ at 324.2 K (E_a_ = E_v_ + 0.41 eV, N_t_ = 1.32×10¹⁵ cm⁻³)

In contrast, MAPbI_3_:PLL exhibited only E_1_ at 348.6 K (E_a_ = E_c_ – 0.88 eV, N_t_ = 1.28×10¹⁵ cm⁻³). The absence of H_1_ indicates that PLL suppresses hole-related traps, while the deeper E_1_ suggests enhanced electron localization, likely linked to nonlinear photoresponse behaviors.

**Section 5.** Optical Characterization


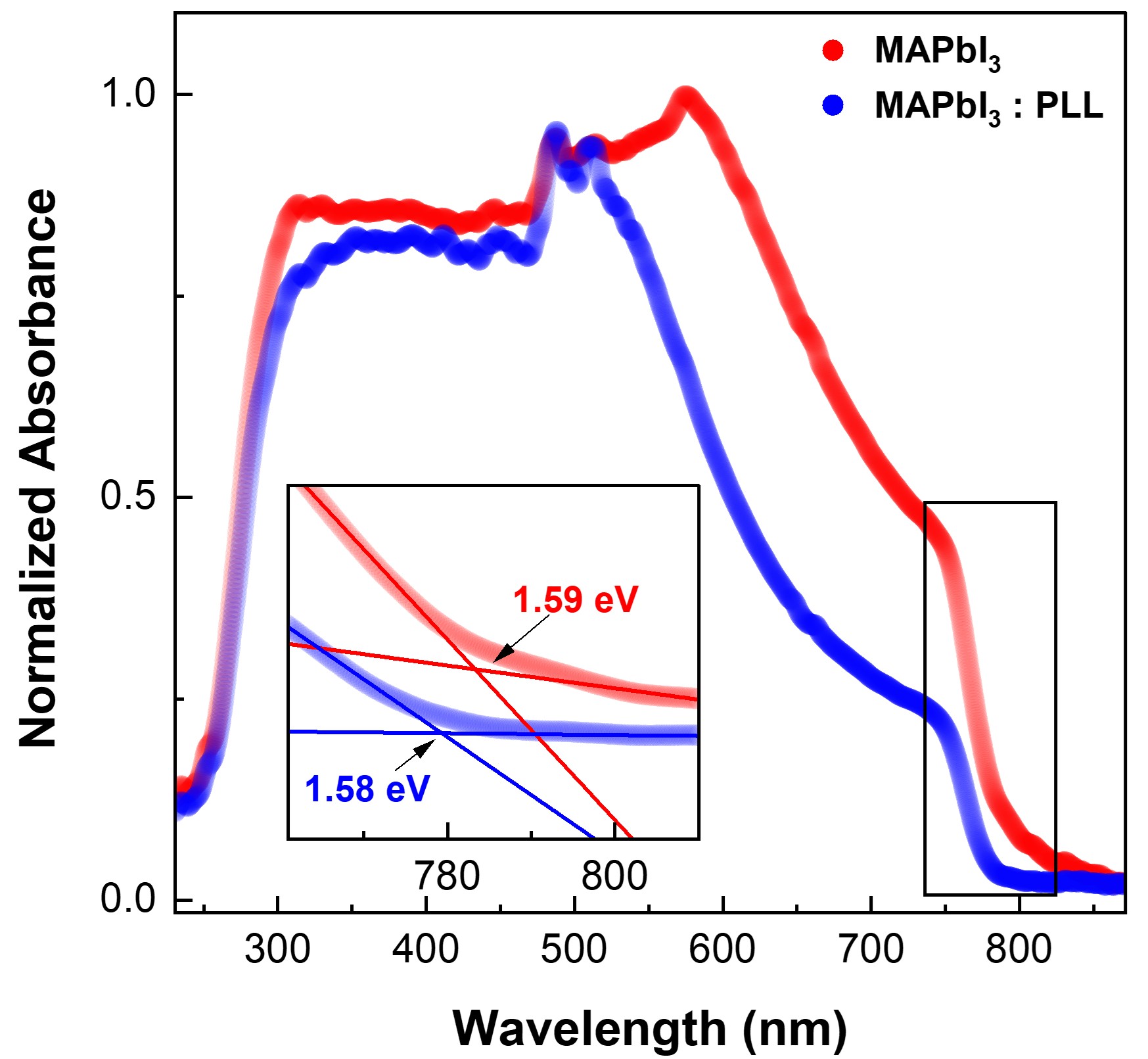


**Figure S5.** Normalized absorbance spectra with slightly reduced intensity for MAPbI₃:PLL, while the bandgap remains unchanged (~1.58 eV)

Absorbance spectra showed reduced intensity in the 500–700 nm range, suggesting enhanced nonradiative recombination via trap states,^[1]^ while the optical bandgap remained nearly unchanged (~1.58 eV).

**Section 6.** Grazing Incidence Wide Angle X-ray Scattering (GIWAXS) Analysis


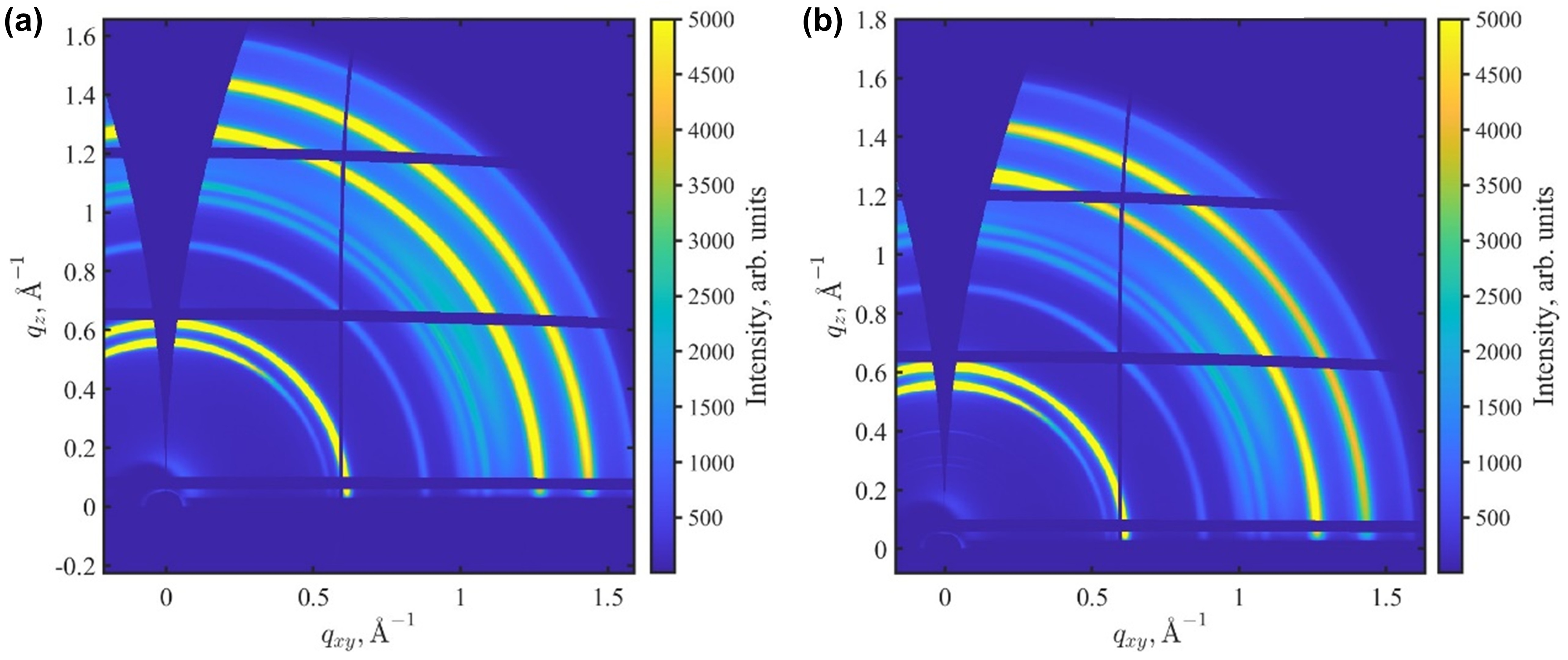


**Figure S6.** Grazing Incidence Wide Angle X-ray Scattering (GIWAXS) data (a) MAPbI_3_ and (b) MAPbI_3_ : PLL

GIWAXS analysis shows that both pristine and PLL-doped MAPbI_3_ films exhibit isotropic diffraction rings, indicating randomly oriented grains with preserved overall crystal structure (Figure S6a and 6b). However, the PLL-doped sample shows a reduced diffraction intensity at q ≈ 1.4 Å^-1^, implying slightly decreased crystallinity due to localized lattice distortion consistent with trap states identified via DFT and DLTS.

The prominent diffraction ring at q ≈ 1.4 Å^-1^ corresponds to the (110) plane of tetragonal MAPbI_3_ with d-spacing of approximately 4.5 Å. Additional rings at q ≈ 0.9 Å^-1^ and q ≈ 0.6 Å^-1^ match the (112) and (202) planes, respectively.

**Section 7. Surface SEM Image**


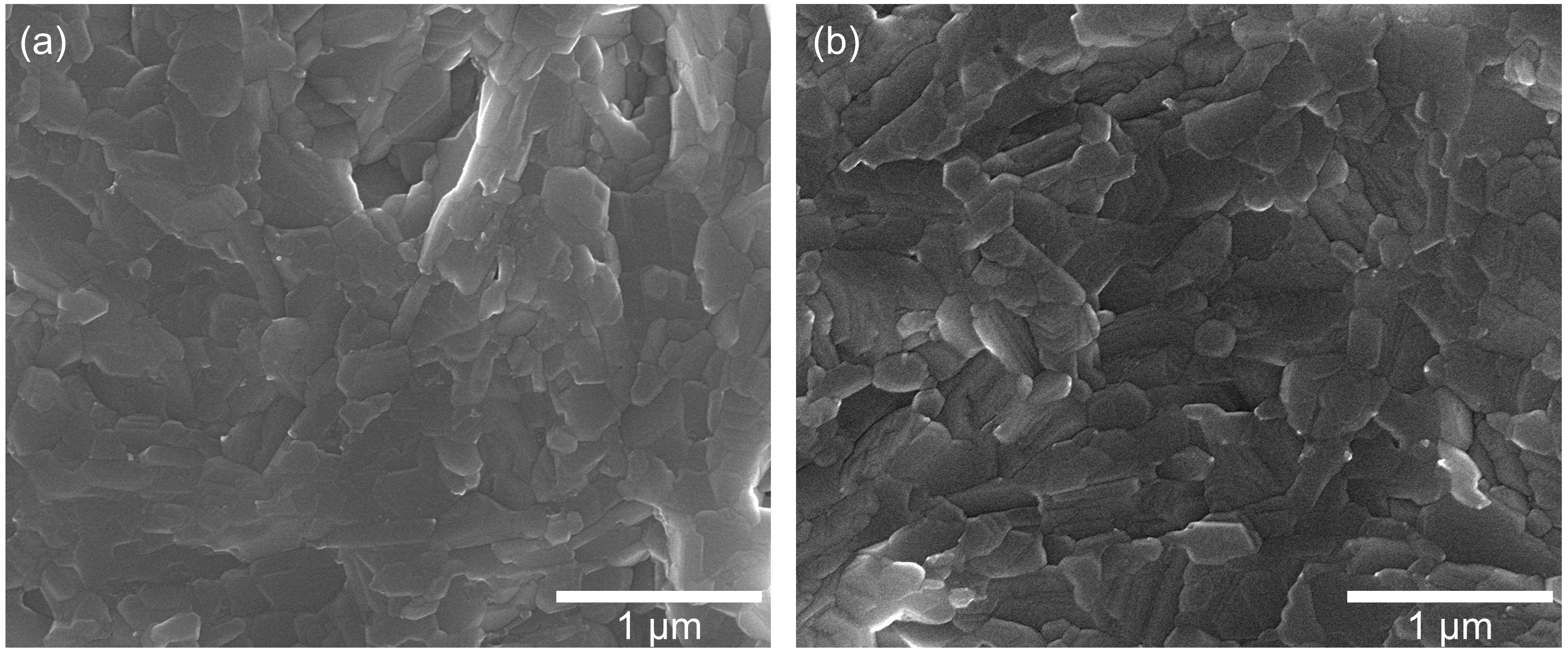


**Figure S7.** Surface SEM images of (a) pristine perovskite and (b) polymer-blended perovskite films.

**Section 8.** Density Functional Theory (DFT) Simulation


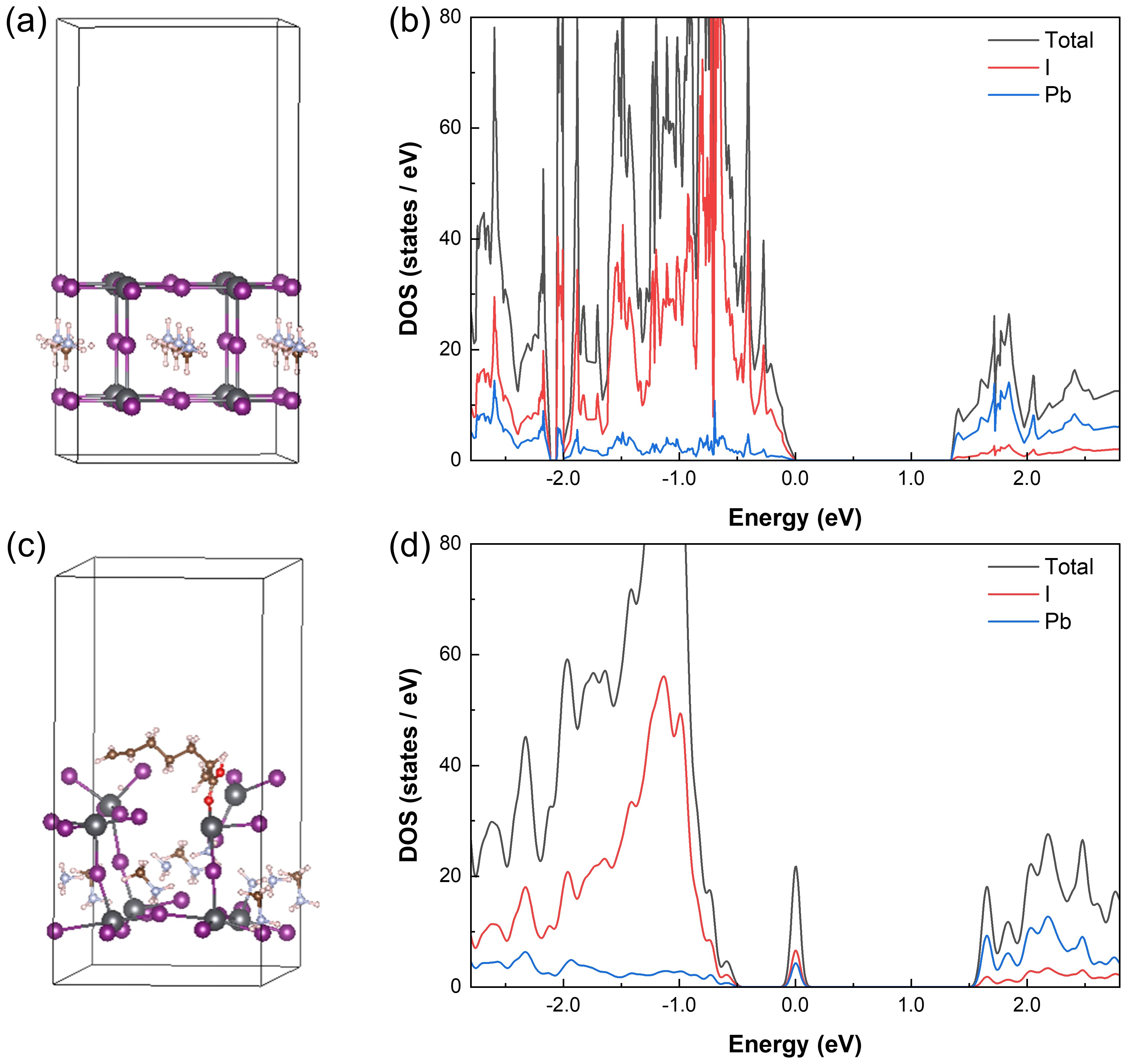


**Figure S8.** (a) Structural model of pristine MAPbI_3_ (b) Density of States (DOS) and projected DOS (I, Pb) of pristine MAPbI_3_ (c) Structural model of PLL(Poly-l-lysine) doped MAPbI_3_ (d) Density of States (DOS) and projected DOS (I, Pb) of PLL doped MAPbI_3_.

Density Functional Theory (DFT) simulations show that pristine MAPbI_3_ (Figures S8a, b) exhibits a density of states (DOS) near the band edges, with no significant in-gap states. After PLL doping (Figures S8c, d), localized states appear near the conduction and valence band edges. These states are sharp and concentrated, and notably accompanied by an increased contribution from iodine orbitals in the projected DOS, suggesting that PLL doping introduces iodine-related lattice distortions.

**Section 9.** Spatial Mapping of Photovoltage Response

***
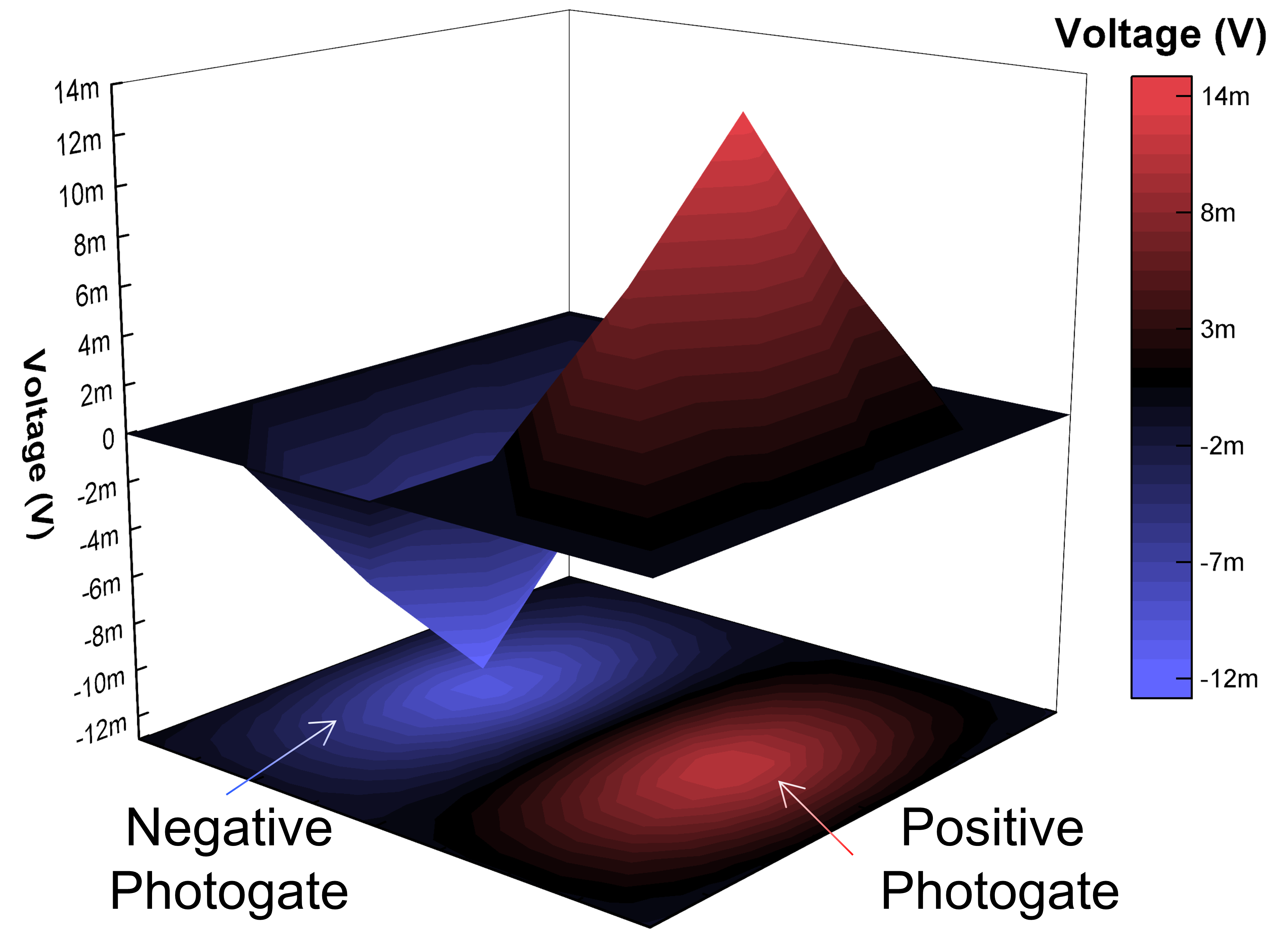
***

**Figure S9.** Spatial mapping of the photovoltage response as a function of light position (x and y directions) across the positive (Au) and negative (glass) photogate regions. The illumination intensity was fixed at 30 mW. The open-circuit voltage peaks at the center of each electrode and gradually decreases with distance, confirming spatially resolved and direction-sensitive photoresponse.

**Section 10.** Device-to-Device Reproducibility


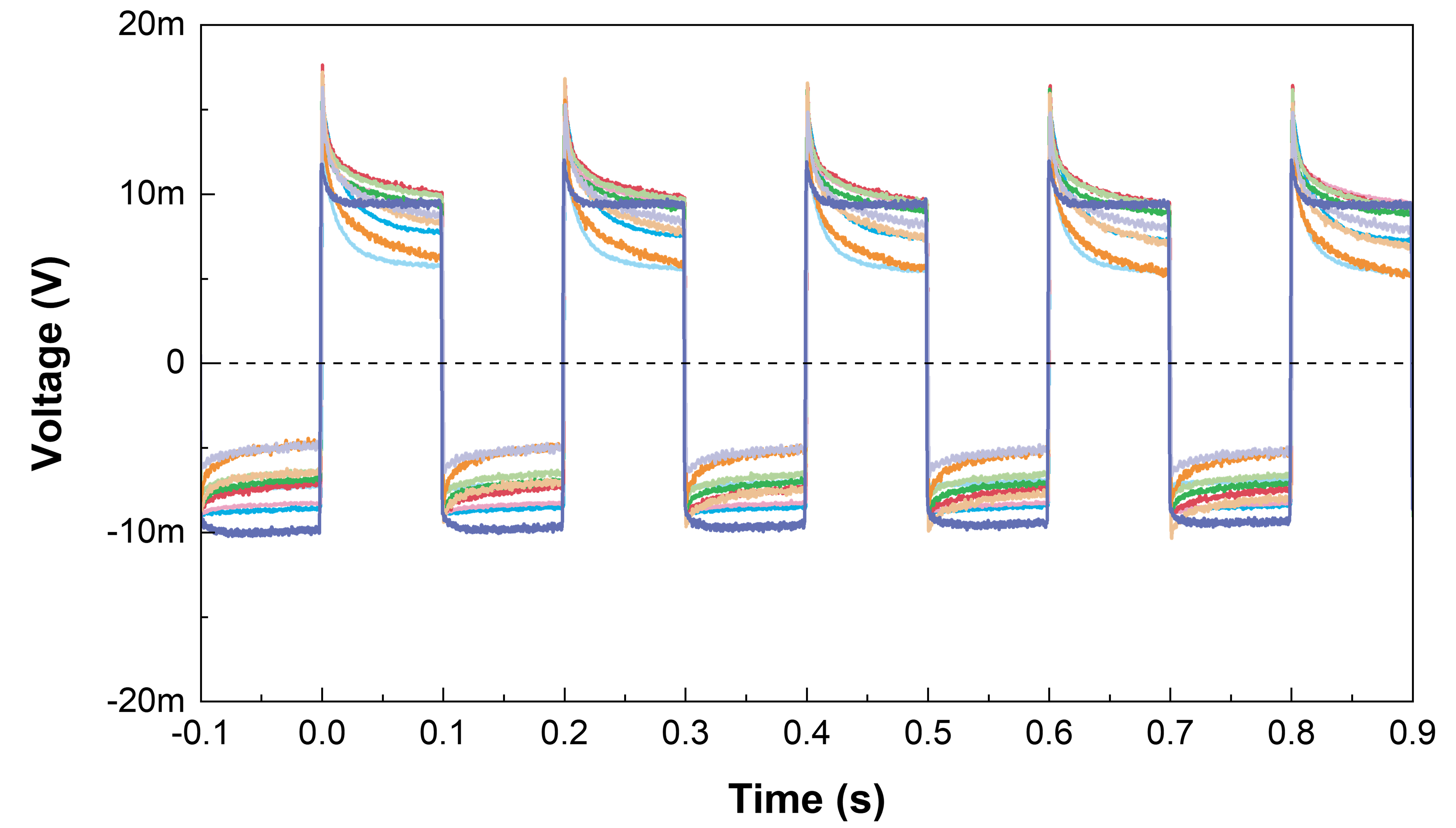


**Figure S10.** Open-circuit voltage responses of ten devices under alternating illumination of the negative and positive photogates.

**Section 11.** Endurance Test Under Repeated Illumination


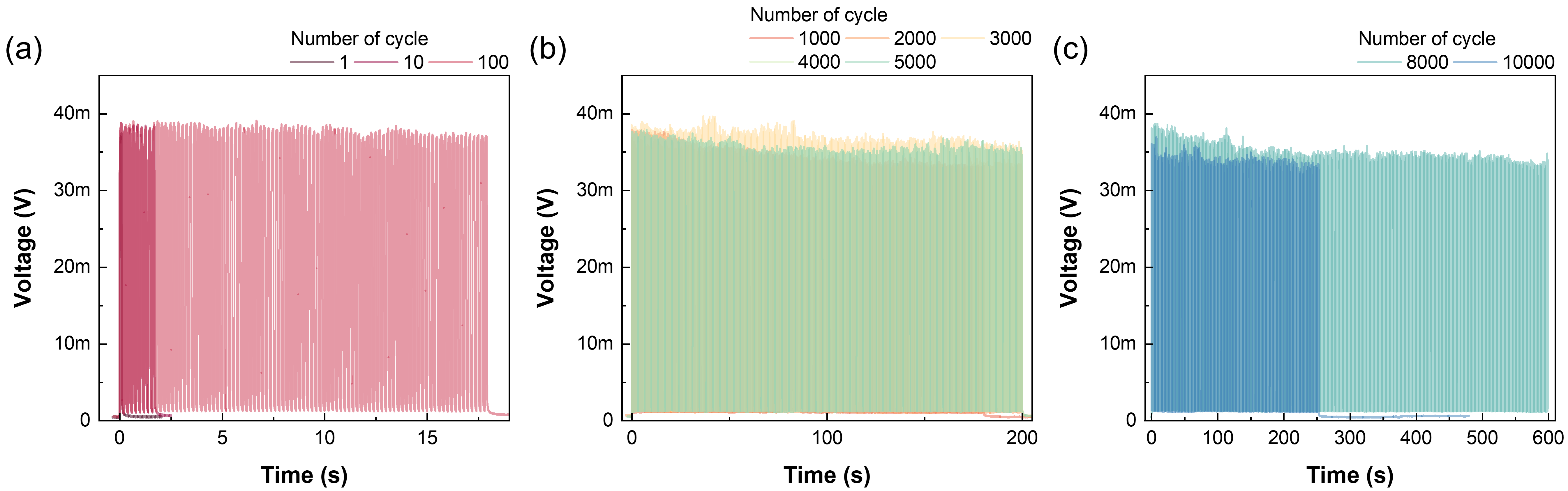


**Figure S11.** Endurance test under repeated 100 ms on/off light pulses. (a) Output voltages measured from cycles 1 to 100. (b) Cycles 1000 to 5000. (c) Output voltage at cycle 10000.

**Section 12.** Impedance Spectroscopy of the Negative Photogate


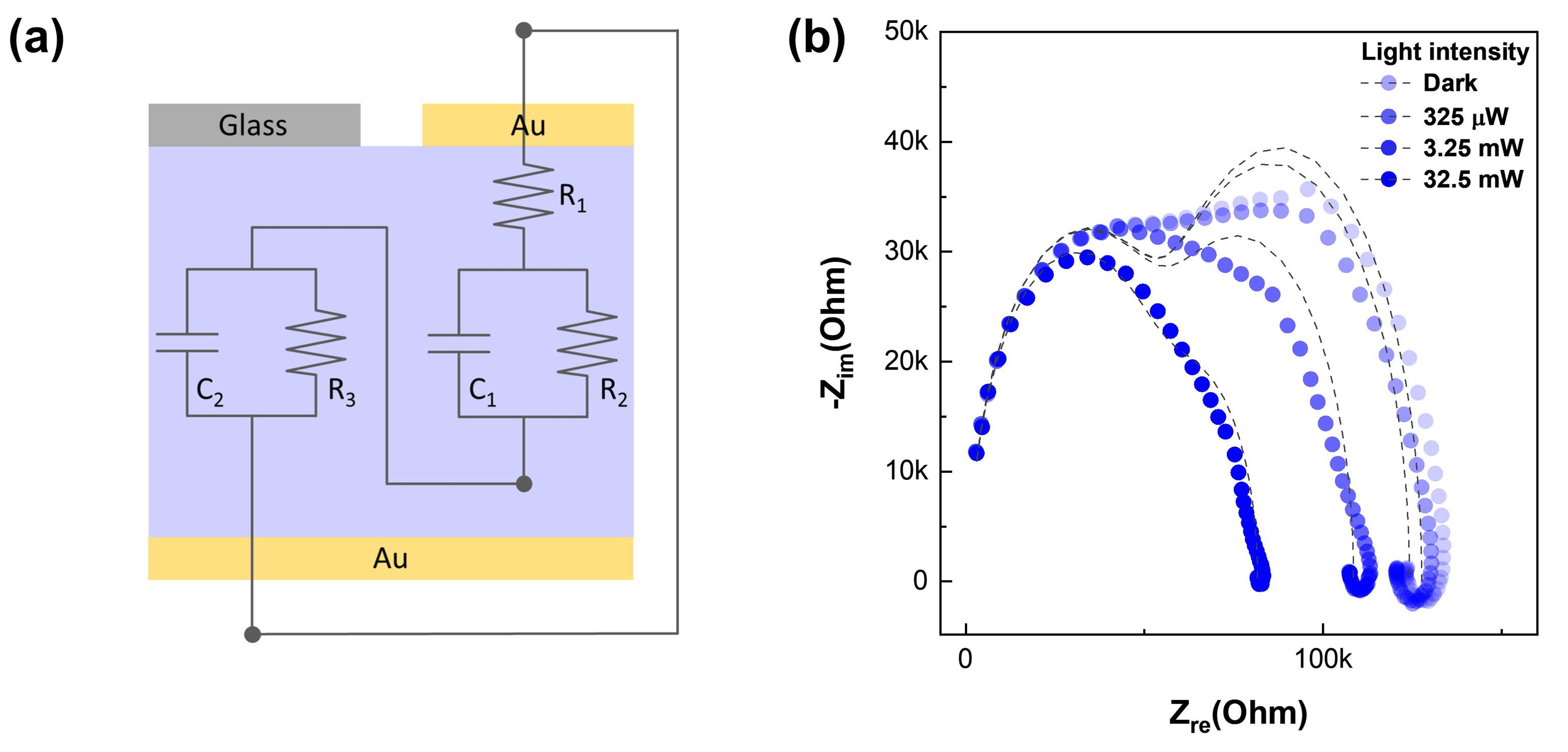


**Figure S12.** (a) Schematic of the impedance measurement setup and equivalent circuit model for the negative photogate. (b) Nyquist plots under illumination (0 to 32.5 mW), fitted using the equivalent circuit (dotted lines). The resistance R_3_ component notably decreases with increasing light intensity, indicating enhanced charge extraction toward the bottom Au electrode near the glass substrate. Other circuit parameters remained largely unchanged, consistent with trap-modulated carrier dynamics.^[2]^

**Section 13.** Impedance Spectroscopy of the Positive Photogate


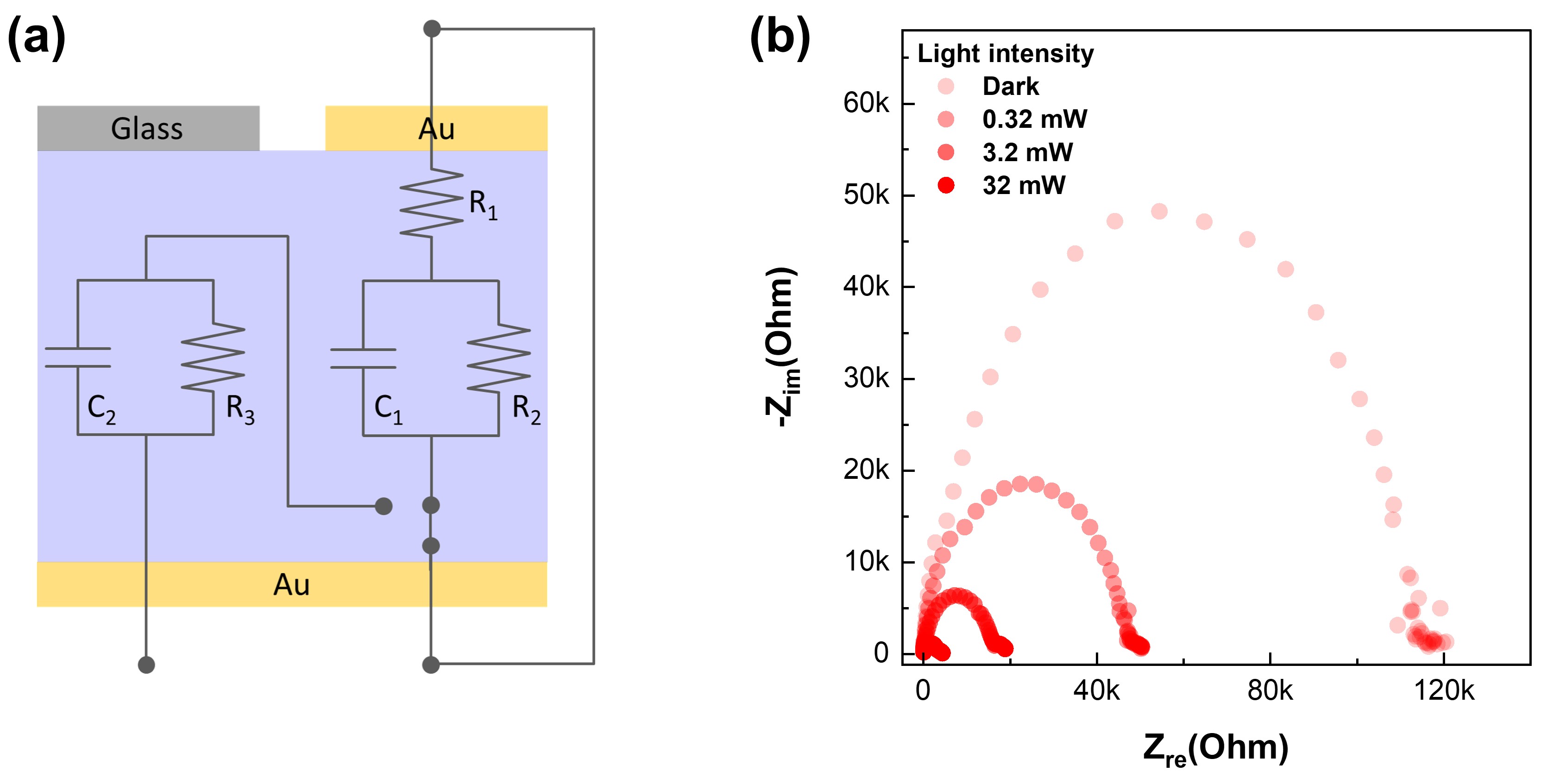


**Figure S13.** (a) Schematic of the impedance measurement setup and equivalent circuit model for the positive photogate. (b) Nyquist plots measured under increasing light intensities (0–32 mW) applied to the positive photogate. The semicircle radius in the Nyquist plots decreases with higher illumination, indicating reduced resistance and enhanced carrier extraction efficiency under strong light exposure. Fitting with an equivalent RC circuit model confirms a significant decrease in charge transport resistance, consistent with improved photocurrent generation efficiency.^[3]^

**Section 14.** Illumination Conditions for Logic Operations

| Logic gate | Modulate (Always on) | | Input A and B | | |
| --- | --- | --- | --- | --- | --- |
|  | Photogate | Intensity  (W/cm^2^) | Photo gate | Intensity (W/cm^2^) | Pulse duration  (ms) |
| YES | N-gate | 0.828 | P-gate | 1.146 | 10 |
| AND | N-gate | 0.828 | P-gate | 0.955 | 10 |
| OR | N-gate | 0.828 | P-gate | 1.465 | 10 |
| NAND | P-gate | 1.465 | N-gate | 1.019 | 10 |
| NOR | P-gate | 1.465 | N-gate | 1.783 | 10 |
| NOT | P-gate | 1.465 | N-gate | 1.019 | 10 |
| XNOR | N-gate | 0.446 | P-gate | 0.701 | 1 |
| XOR | P-gate | 0.796 | N-gate | 0.446 | 1 |

**Table S2.** Detailed illumination conditions for each logic operation shown in Figure 4. All light inputs were delivered at a fixed wavelength of 450 nm through a 105 μm diameter optical fiber.

**Section 15.** Endurance of Light-Driven Nonlinear Logic Operations


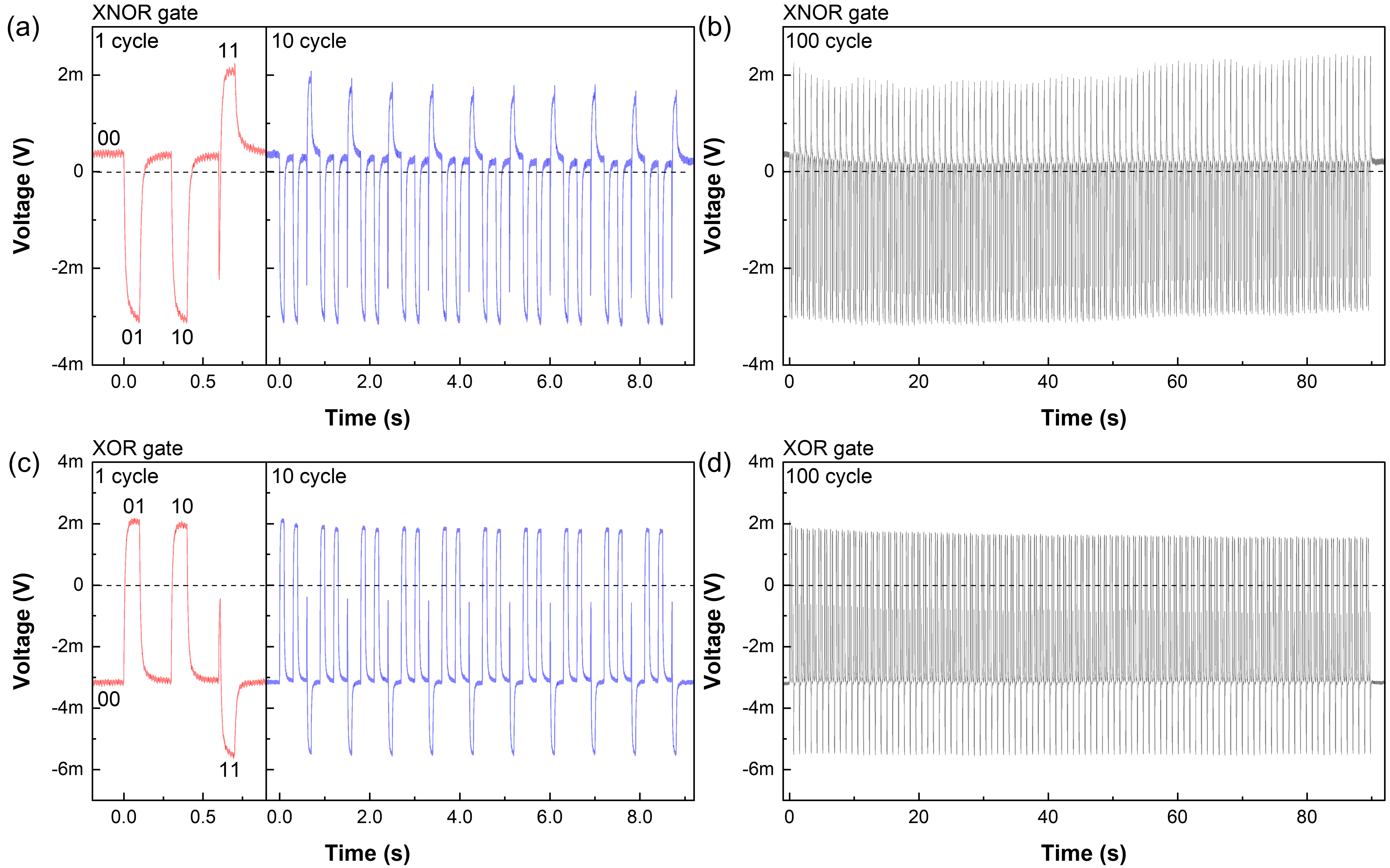


**Figure S14.** Endurance performance of light-driven nonlinear logic operations. (a) XNOR logic outputs at cycles 1 and 10. (b) XNOR operation for 100 switching cycles. (c) XOR logic outputs at cycles 1 and 10. (d) XOR operation for 100 switching cycles.

**Section 16.** Sequential and Dynamic Switching of Logic Operations


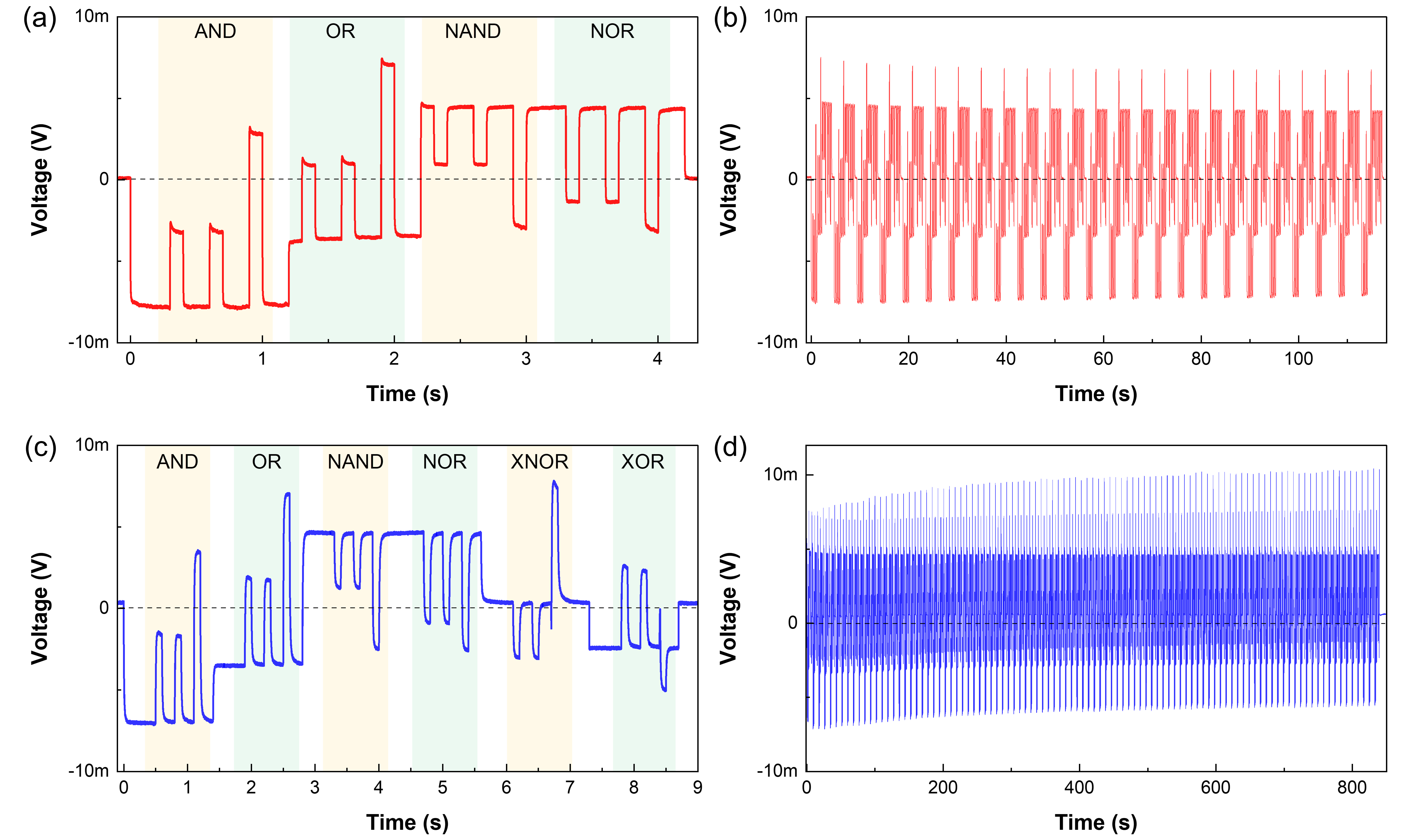


**Figure S15.** (a) Sequential switching operation of linear logic gates including AND, OR, NAND, and NOR. (b) Repeated operation of the same linear sequence over 25 cycles. (c) Dynamic switching among linear and nonlinear logic gates, in the order of AND, OR, NAND, NOR, XNOR, and XOR. (d) Continuous operation of the switching logic sequence sustained for over 800 seconds.

**Section 17.** Analog Signal Separation for Dual-Logic Operations


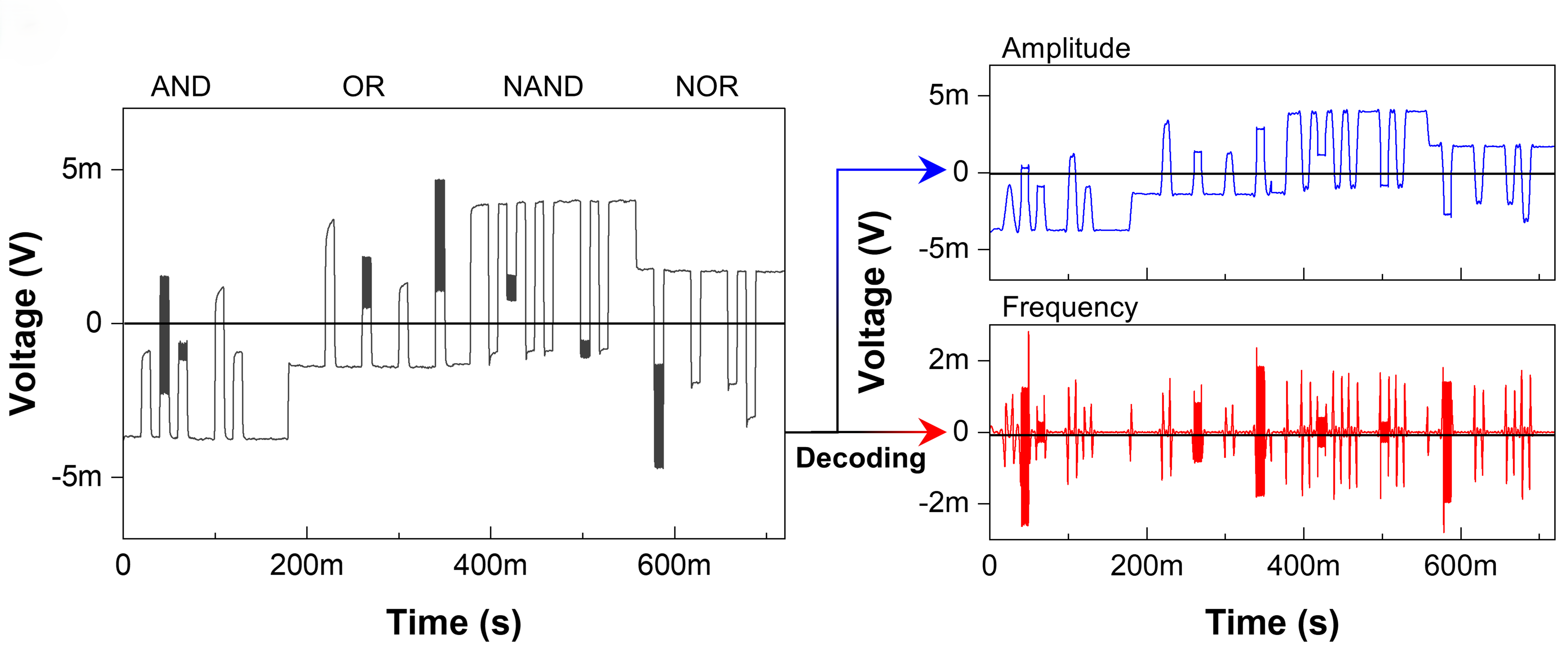


**Figure S16.** (a) Experimental dual-channel signal separation and binarization using the dual-photogate device. A composite optical input containing both amplitude content and temporally modulated components was applied to the device. The measured photovoltage trace is decomposed into parallel amplitude-response (blue) and temporal-frequency–response (red) traces via band-limited demodulation. Each trace is binarized with calibration thresholds to generate channel-specific logic inputs and outputs

**Section 18.** Analog Voltage Maps Prior to Binarization


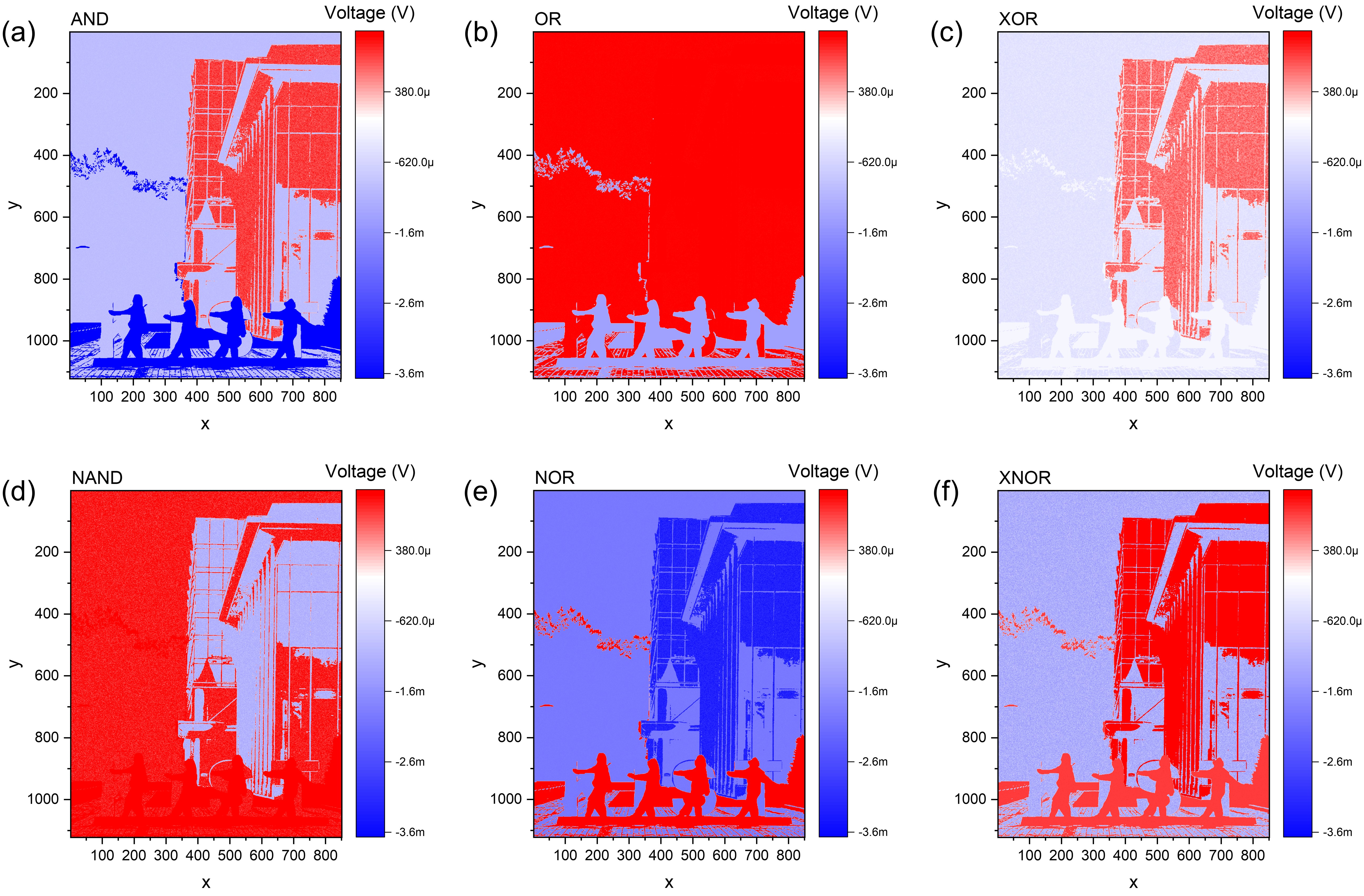


**Figure S17.** Analog voltage maps corresponding to six logic operations applied to a visual scene, based on non-binarized data obtained from the MAPbI_3_:PLL device. Each panel shows the spatial distribution of analog voltage before binarization: (a) AND, (b) OR, (c) XOR, (d) NAND, (e) NOR, and (f) XNOR. Higher voltage regions (red) represent areas tending toward logical 1, and lower voltage regions (blue) represent areas tending toward logical 0.

**Section 19.** Commercial vision sensors and preprocessing strategies

| Category | Device | Vendor | Modality | FrameRate/Latency | Power | Ingress/IP | ref |
| --- | --- | --- | --- | --- | --- | --- | --- |
| LiDAR | AT128 | Hesai | Time-of-Flight LiDAR (Hybrid solid-state) | 10/20 Hz | ≈13.5 W |  | 4 |
|  | OS2-128 | Ouster | Time-of-Flight LiDAR (spinning) | 10/20 Hz | 18–24 W | IP68/IP69K | 5 |
|  | InnovizTwo | Innoviz | Solid-state LiDAR (MEMS) | 10/20 FPS |  |  | 6 |
|  | Iris+ | Luminar | LiDAR (1550 nm) | 1–30 fps (Iris) | ~25 W (Iris) | IP69K (Iris) | 7 |
|  | Vista-X120 Plus | Cepton | LiDAR |  | <18 W |  | 8 |
|  | RS-LiDAR-M1 / M1 Plus | RoboSense | Solid-state LiDAR (2D MEMS) |  | ≈15 W | IP67 / IP6K9K | 9 |
|  | SCALA Gen 3 | Valeo | Automotive LiDAR | 10-20 fps |  |  | 10 |
|  | Aeries II | Aeva | FMCW 4D LiDAR (LiDAR-on-chip) |  |  | IP67 | 11 |
| Radar | ARS540 | Continental | 77 GHz 4D Imaging Radar |  |  |  | 12 |
|  | 4D Imaging/Full-Range Radar | ZF | 77 GHz 4D Imaging Radar |  |  |  | 13 |
|  | Phoenix Perception Radar | Arbe Robotics | 4D Imaging Radar | 20 FPS |  |  | 14 |
|  | S81 RoC | Uhnder | 77–81 GHz PMCW 4D Imaging Radar-on-Chip |  |  |  | 15 |
| Thermal (LWIR) | ADK | Teledyne FLIR | Thermal camera module (Boson core) | 30/60 Hz (9 Hz option) | ~4 W average | IP67; heated window | 16 |
|  | Thermal Ranger (3D) | Owl Autonomous Imaging | 3D Thermal + RGB |  |  |  | 17 |
|  | Boson+ (module) | Teledyne FLIR | Thermal camera core | 30/60 Hz | ~0.5 W |  | 18 |
| Event Camera | EVK4 HD (IMX636) | Prophesee/Sony | Event-based vision sensor | Pixel latency <100 µs (@1000 lux) | ~205 mW |  | 19 |
|  | DVXplorer | iniVation | Event-based camera | Latency <1 ms; 200 µs temporal res |  |  | 20 |
| ToF Depth | blaze-102 | Basler | iToF 3D depth camera (IMX556) | Up to 30 fps |  | IP67 (with housing/cable) | 21 |
|  | O3D303 | ifm | PMD iToF 3D camera | Up to 25 Hz |  |  | 22 |
| Logic Device | OELG device Perovskite OELG | Research prototype | Intensity & frequency-domain separation with integrated logic | μs-scale operation | Zero-bias operation | Not applicable (prototype) | This work |

**Table S3.** Representative specifications of commercially available sensors relevant to autonomous driving.

Table S3 summarizes representative specifications of commercial sensors. LiDAR operates at a few to tens of hertz, ToF depth cameras at tens of fps, thermal modules at 30–60 Hz, and event cameras at μs–ms latency. The OELG exhibits a nonlinear response at the photogate that enables intrinsic thresholding without extra circuits, achieving μs-scale operation and suggesting the potential for reduced latency compared with event cameras. It also operates without external bias and reduces downstream data through front-end masking, showing the potential for lower power consumption. In terms of robustness, although it lacks encapsulation and cannot be directly evaluated by IP standards, the device exhibited stable operation for 10 000 cycles (Figures S13–S15), and light spreading or ambient interference can be mitigated by apertures, diffusers, or band-pass filters.

| ref | Category | Device/Model | Modality | Data Reduction Method | Output Format | Operating Speed / Latency |
| --- | --- | --- | --- | --- | --- | --- |
| 23 | Smart Vision Sensor (AI on-sensor) | Sony IMX500 | RGB | Metadata instead of full frame | Object position/label metadata | Object recognition ~3.1 ms |
| 24 | Event Camera | Sony/Prophesee IMX636 | Event (DVS) | Outputs events only when changes occur | Event stream (polarity, coordinates, timestamp) | Pixel latency <100 µs @1000 lux |
| 25 | Event Camera | Prophesee GenX320 | Event (DVS) | Frameless event-based | Event stream | µs-level timestamp |
| 26 | Event Camera | iniVation DVXplorer | Event (DVS) | Outputs events only when changes occur | Event stream | Temporal res 200 µs, latency <1 ms; up to 165 MEvents/s |
| 27 | Event Camera | CelePixel CeleX‑V | Event/Frame Hybrid | Event-based output | Event/Frame | High-speed mode up to tens of thousands FPS (see product) |
| 28 | Focal-plane Processing (PPA) | SCAMP‑5d Vision System | Focal-plane SIMD | On-sensor processing, summary output only | Feature map/Results | Core ops at several kFPS |
| 29 | Stereo Depth (On-camera processing) | Nerian Scarlet 3D Camera | Stereo | Depth/Disparity output | Depth/Disparity map | Up to 120 FPS |
| 30 | Stereo Depth (On-camera processing) | Intel RealSense D435/D435i | Active Stereo | Depth map output (computed on-board) | Depth map | Up to 90 FPS |
| 31 | ToF Depth (On-sensor/module) | Basler blaze | iToF | Depth output | Depth map | 30 FPS |
| 32 | ToF Image Sensor | Infineon REAL3 IRS2381C | iToF | Depth, Amplitude | Depth map, amplitude image | Real-time operation |
| 33 | ToF Multi-zone Sensor | ST VL53L5CX | Direct ToF (SPAD) | Zone distance output | 4×4 or 8×8 zone distance | Up to 60 Hz |
| 34 | Optical Flow Sensor | PixArt PMW3901 | Optical Flow | Low-dimensional vectors instead of full image | Δx, Δy, quality |  |
| 35 | Ultra-low-power Always-On Camera | Himax HM01B0 | Monochrome | Motion detection with interrupt | Frame/Event interrupt | 30-120 FPS |
| 36 | Ultra-low-power Always-On Camera | Himax HM0360 + WiseEye | RGB/Monochrome | Programmable with event interrupt | Frame/AI result output | Low-power standby with interrupt wake-up |
| 37 | On-camera AI (SoC) | Luxonis OAK‑D S2 (Myriad X) | RGB + Stereo | Outputs full frames | Depth/Detection results | Real-time inference/stereo (see product spec) |
| 38 | On-camera AI | Teledyne FLIR Firefly DL | RGB | Outputs full frames | Detection metadata | Real-time inference |
| 39 | On-camera AI (SoC) | Hailo‑15 Smart Camera SoC | NPU SoC | Outputs full frames | Detection/Analysis results | Up to 20 of TOPS real-time |
| 40 | On-camera AI (SoC) | Ambarella CV28M | AI Camera SoC | Smart encoding/Metadata | Analysis results/H.264 etc. | Real-time |
| 41 | Onboard ISP/Preprocessing | Allied Vision Alvium 1800 Series | RGB/Mono | Reduces host CPU load/bandwidth | Preprocessed frame | Camera framerate |
| 42 | Edge TPU Board (with Camera) | Google Coral Dev Board | Edge TPU + Camera | Full image on device inference | Detection results/Metadata | Real-time inference |
| 43 | Microvision Camera Board | OpenMV Cam H7 Plus | Microcontroller Camera | Onboard preprocessing with simple vision algorithms | Frame results | 20-50 FPS |
| 44 | Microvision Camera Board | JeVois‑Pro | Smart USB Camera | Optional result-only mode | Detection/Serial metadata | Real-time |
| 45 | Edge AI Camera Module | Sipeed Maix (K210) AI Camera | KPU + Camera | Optional results via serial metadata | Detection, bounding box | Real-time |
| 46 | ToF/Depth (Smartphone) | Samsung ISOCELL Vizion 63D | iToF | Depth map/image data | Depth/Distance output | Up to 60 FPS |
| This  work | Optoelectronic Logic Gate | OELG (This work) | Amplitude + Temporal modulation | Intrinsic thresholding via nonlinear bipolar photoresponse | ROI mask | μs-scale operation; |

**Table S4.** Comparison of representative commercial and research vision sensors highlighting their data reduction strategies.

Table S4 summarizes representative data reduction strategies of commercial and research sensors. On-sensor AI devices operate at ~3 ms, ToF modules at 30–60 fps, PPA at several thousand fps, and event cameras at the μs–ms scale. The OELG, while structurally different, can perform comparable functions by using nonlinear and bipolar photoresponse to perform eight logic operations within a single device at the μs scale. It operates at zero external bias with power consumption estimated to remain within tens of mW even under modulated illumination, and conceptually can reduce downstream load through front-end masking. In terms of robustness, event cameras are sensitive to flicker and thresholds, and PPA is limited in background suppression, while the OELG enables threshold-free polarity switching with a very high on/off ratio and can employ modulation light for noise reference. Full robustness tests under real-world conditions such as light spreading or ambient interference remain beyond the scope of this work.

| - **Ref** | **Device/System** | **Principle/Preprocessing Role** | **Key Metrics/Notes** | **Applications** |
| --- | --- | --- | --- | --- |
| 47 | Infineon REAL3™ ToF Imager | Lock-in pixel (photonic mixer) for phase/amplitude acquisition | Up to tens of MHz modulation (product dependent) | Mobile/automotive 3D sensing |
| 49 | SCAMP-5 Pixel Processor Array | Per-pixel processor/bit-register logic (OR/NOR/NOT etc.) | 256×256 PPA | On-sensor preprocessing |
| 50 | Dynamic Vision Sensor (DVS) | Pixel-level comparator threshold (asynchronous event) | 120 dB DR, μs latency | High-speed tracking/industry |

**Table S5.** Examples of sensor-level preprocessing devices.

Table S5 summarizes representative preprocessing devices and compares them with the OELG. The Infineon REAL3 ToF Imager detects phases by mixing reflections with a reference, conceptually similar to how the OELG can respond to fast modulation under mixed illumination. The SCAMP-5 PPA performs logic through per-pixel processors and registers, while the OELG executes up to eight logic operations in a single unit, suggesting the potential to reduce power and area. The DVS outputs μs-latency events via per-pixel comparators but requires heavy backend buffering, whereas the OELG enables XOR and XNOR execution through nonlinear polarity switching at a natural 0 V threshold in our proof-of-concept demonstration.

**References**

[1] Y. Wen, T. Zhang, X. Wang, T. Liu, Y. Wang, R. Zhang, M. Kan, L. Wan, W. Ning, Y. Wang, D. Yang, *Nat. Commun*. **2024**, 15, 7085, <https://doi.org/10.1038/s41467-024-51551-y.>

[2] J. R. Macdonald, *Ann. Biomed. Eng.* **1992**, 20, 289–305, <https://doi.org/10.1007/BF02368532.>

[3] E. Ghahremanirad, O. Almora, S. Suresh, A. A. Drew, T. H. Chowdhury, A. R. Uhl, *Adv. Energy Mater.* **2023**, 13, 2204370, <https://doi.org/10.1002/aenm.202204370.>

[4] Hesai, *AT128E2X 128-Channel Hybrid Solid-State LiDAR Manual*, Hesai AT128 Product Manual, https://manuals.plus/hesai/at128e2x-128-channel-hybrid-solid-state-lidar-manual (accessed: Sep. 10, 2025).

[5] Ouster, *OS-Overview*, Ouster OS LiDAR System Overview Page, https://ouster.com/os-overview (accessed: Sep. 10, 2025).

[6] Innoviz, *InnovizTwo Product Page*, Innoviz Two – Automotive LiDAR, https://innoviz.tech/innoviztwo (accessed: Sep. 10, 2025).

[7] Luminar Technologies, *Technology* — Luminartech Technology Page, https://www.luminartech.com/technology (accessed: Sep. 11, 2025).

[8] Cepton, *Vista-X120 Plus Product Page*, Cepton Vista-X120 Plus — Automotive LiDAR, https://www.cepton.com/vista-x120-plus/reveal (accessed: Sep. 11, 2025).

[9] RoboSense, RS-LiDAR-M1P User Guide, RoboSense Documentation, https://github.com/RoboSense-LiDAR/rslidar_sdk (accessed: Sep. 11, 2025).

[10] Valeo, *Long-Range Lidar Sensors:* SCALA Gen 3 Catalogue, https://www.valeo.com/en/catalogue/cda/long-range-lidar-sensors-valeo-scala-gen-3/ (accessed: Sep. 11, 2025)

[11] Aeva, *Aeries II: the world’s first 4D LiDAR with camera-level resolution*, Press Release, https://www.aeva.com/press/aeva-introduces-aeries-ii-the-worlds-first-4d-lidar-with-camera-level-resolution (accessed: Sep. 11, 2025)

[12] Continental; Xilinx, *Continental ARS540 Powered by Xilinx – 77 GHz 4D Imaging Radar Overview*, Technical Document, September 22, 2020. (accessed: Sep. 11, 2025).

[13] ZF, *ZF 4D Imaging Radar Launch Press Release*, https://press.zf.com/press/en/releases/release_48960.html (accessed: Sep. 11, 2025).

[14] Arbe Robotics, *Phoenix Perception Radar Product Page*, https://arberobotics.com/perception-radar/ (accessed: Sep. 11, 2025).

[15] Uhnder, *S81 Radar-on-Chip (RoC) Product Technical Brief*, Version 1.0, April 2024.

[16] Teledyne FLIR, *FLIR ADK Thermal Imager Data Sheet (Boson-based)*, Rev. 09/04/2025, https://www.flir.com/adk (accessed: Sep. 11, 2025).

[17] Owl Autonomous Imaging, *Monocular 3D Thermal Ranger™ Computer Vision for ADAS & Autonomous Vehicles – Press Release*, Owl AI, Jan. 3, 2023, https://www.owlai.us/about-owl-ai/news/press-releases/press-release-owl-autonomous-imaging-launches-monocular-3d-thermal-rangertm-computer-vision-for-adas-autonomous-vehicles/ (accessed: Sep. 11, 2025).

[18] Teledyne FLIR, *Boson+ OEM LWIR Camera Module Product Page*, FLIR OEM, https://oem.flir.com/ko-kr/products/boson-plus/?segment=oem&vertical=lwir (accessed: Sep. 11, 2025).

[19] Prophesee; Sony, *Event-Based Sensor IMX636 Product Page*, Prophesee–Sony, https://www.prophesee.ai/event-based-sensor-imx636-sony-prophesee/ (accessed: Sep. 11, 2025).

[20] iniVation AG, *DVXplorer User Guide and Specifications*, Release 2025-08-05, https://docs.inivation.com/_static/hardware_guides/dvxplorer.pdf (accessed: Sep. 11, 2025).

[21] Basler AG, *Basler blaze-102 ToF Camera Product Page*, https://www.baslerweb.com/en-us/shop/blaze-102 (accessed: Sep. 11, 2025)

[22] ifm electronic, *O3D303 3D ToF Camera Product Page*, https://www.ifm.com/us/en/product/O3D303 (accessed: Sep. 11, 2025)

[23] Sony Corporation, *Sony IMX500 Smart Vision Sensor Page*, Developer.Sony, https://developer.sony.com/imx500 (accessed: Sep. 11, 2025)

[24] Prophesee; Sony, Event-Based Sensor IMX636 Product Page, Prophesee–Sony, https://www.prophesee.ai/event-based-sensor-imx636-sony-prophesee/ (accessed: Sep. 11, 2025)

[25] Prophesee, Event-Based Sensor GenX320 Product Page, Prophesee, https://www.prophesee.ai/event-based-sensor-genx320/ (accessed: Sep. 11, 2025)

[26] iniVation AG, DVXplorer Product Page/Specifications, https://docs.inivation.com/hardware/current-products/dvxplorer (accessed: Sep. 11, 2025)

[27] S. Chen, M. Guo, *Live Demonstration: CeleX-V – A 1M Pixel Multi-Mode Event-Based Sensor*, Demonstration Paper, Nanyang Technological University & CelePixel Technology, Singapore, 2018.

[28] J. Chen, S. J. Carey, P. Dudek, *Scamp5d Vision System and Development Framework: Demonstration Paper,* Proc. Int. Conf. Distributed Smart Cameras (ICDSC), Eindhoven, Netherlands, Sept. 2018, pp. 1–2. https://doi.org/10.1145/3243394.3243698

[29] Nerian Vision Technologies, *Nerian Scarlet 3D Depth Camera Product Page, SodaVision*, https://www.sodavision.com/product/nerian-scarlet-3d-depth-camera/ (accessed: Sep. 11, 2025)

[30] Intel Corporation, *Intel® RealSense™ Depth Camera D400 Series (D415, D435, D435i) Product Family Datasheet, Rev. 005*, Jan. 2019. https://www.intel.com/design/literature.htm (accessed: Sep. 11, 2025)

[31] Basler AG, *Basler blaze ToF Camera (IMX556) Datasheet*, 2023. https://www.baslerweb.com/en-us/shop/blaze-102 (accessed: Sep. 11, 2025)

[32] Infineon Technologies, *REAL3™ IRS2381C 3D Time-of-Flight Image Sensor, Product Brief*, Order No. B132-I0738-V1-7600-EU-EC, Feb. 2019. https://www.infineon.com/real3 (accessed: Sep. 11, 2025)

[33] STMicroelectronics, *VL53L5CX: Time-of-Flight 8×8 Multizone Ranging Sensor with Wide FoV, Datasheet DS13754 Rev. 13*, Sept. 2024. https://www.st.com/en/product/vl53l5cx (accessed: Sep. 11, 2025)

[34] STMicroelectronics, *VL53L7CX: Time-of-Flight 8×8 Multizone Ranging Sensor with 90° FoV, Datasheet DS13865 Rev. 8*, Sept. 2024. https://www.st.com/en/product/vl53l7cx (accessed: Sep. 11, 2025)

[35] Himax, *HM01B0 Ultra-Low-Power Always-On Sensor Product Page*, Himax Always-On Vision Sensors, https://www.himax.com.tw/ko/products/cmos-image-sensor/always-on-vision-sensors/hm01b0/ (accessed: Sep. 11, 2025)

[36] Himax WiseEye Solutions, *WiseEye AI Sensing Solutions Product Page*, Himax, https://www.himax.com.tw/products/wiseeye-ai-sensing/wiseeye-solutions/ (accessed: Sep. 11, 2025)

[37] Luxonis, *OAK-D S2 Camera Module Product Page*, Luxonis OAK-D S2, https://shop.luxonis.com/products/oak-d-s2?variant=42455432233183 (accessed: Sep. 11, 2025)

[38] Teledyne Vision Solutions, *Firefly DL Smart Camera Product Page*, https://www.teledynevisionsolutions.com/products/firefly-dl/?segment=iis&vertical=machine+vision (accessed: Sep. 11, 2025)

[39] Ambarella, *CV28M Computer Vision SoC Product Brief*, Ambarella International LP, Nov. 2022 .

[40] Hailo Technologies, *Hailo-15 AI Vision Processor Product Brief*, Hailo Technologies Ltd., Jul. 2023

[41] Allied Vision, *Alvium 1800 USB Camera with High-Performance Sony Sensors, Digi-Key Product Highlight*, https://www.digikey.com/en/product-highlight/a/allied-vision/alvium-1800-usb-camera-with-high-performance-sony-sensors (accessed: Sep. 11, 2025)

[42] Google Coral, *Coral Development Board (Edge TPU) Product Page*, https://coral.ai/products/dev-board (accessed: Sep. 11, 2025)

[43] OpenMV, *OpenMV Cam H7 Plus Product Page*, https://openmv.io/products/openmv-cam-h7-plus (accessed: Sep. 11, 2025)

[44] JeVois Inc., *JeVois Pro Deep Learning Smart Camera Product Page*, https://www.jevoisinc.com/products/jevois-pro-deep-learning-smart-camera (accessed: Sep. 11, 2025)

[45] Sipeed, *MaixPy DNN (K210) Documentation*, Sipeed Wiki, https://wiki.sipeed.com/soft/maixpy/en/dnn/index (accessed: Sep. 11, 2025)

[46] Samsung Semiconductor, *ISOCELL Vizion 63D iToF Sensor Product Page*, https://semiconductor.samsung.com/image-sensor/tof-sensor/isocell-vizion-63d/ (accessed: Sep. 11, 2025)

[47] Infineon Technologies AG, *REAL3™ Image Sensor IRS2381C—Product Brief, v01.00*, 2019. https://www.infineon.com/dgdl/Infineon-IRS2381C_ProductBrief_v01.00.pdf (accessed: September 9, 2025).

[48] Basler AG, *blaze-101 3D Time-of-Flight Camera—Product Page*, 2025. https://www.baslerweb.com/en/shop/blaze-101/ (accessed: September 9, 2025).

[49] P. Dudek, T. Richardson, L. Bose, S. J. Carey, J. Chen, C. Greatwood, Y. Liu, W. Mayol-Cuevas, *Sci. Robot*. 2022, *7*, eabl7755. https://doi.org/10.1126/scirobotics.abl7755

[50] P. Lichtsteiner, C. Posch, T. Delbrück, *IEEE J. Solid-State Circuits* 2008, *43*, 566–576. https://doi.org/10.1109/JSSC.2007.914337

`
